# Evaluation of a Human T Cell-Targeted Multi-Epitope Vaccine for Q Fever in Animal Models of *Coxiella burnetii* Immunity

**DOI:** 10.3389/fimmu.2022.901372

**Published:** 2022-05-16

**Authors:** Ann E. Sluder, Susan Raju Paul, Leonard Moise, Christina Dold, Guilhem Richard, Laura Silva-Reyes, Laurie A. Baeten, Anja Scholzen, Patrick M. Reeves, Andrew J. Pollard, Anja Garritsen, Richard A. Bowen, Anne S. De Groot, Christine Rollier, Mark C. Poznansky

**Affiliations:** ^1^ Vaccine and Immunotherapy Center, Massachusetts General Hospital, Boston, MA, United States; ^2^ EpiVax, Inc., Providence, RI, United States; ^3^ Oxford Vaccine Group, Department of Paediatrics, The National Institute for Health Research (NIHR) Oxford Biomedical Research Centre, University of Oxford, Oxford, United Kingdom; ^4^ Department of Biomedical Sciences, Colorado State University, Fort Collins, CO, United States; ^5^ Innatoss Laboratories B.V., Oss, Netherlands

**Keywords:** Q fever, Coxiella burnetii, ChAdOx2, multi-epitope vaccine, modified vaccinia Ankara (MVA), interferon gamma (IFNγ), T cell vaccine, non-human primate (NHP)

## Abstract

T cell-mediated immunity plays a central role in the control and clearance of intracellular *Coxiella burnetii* infection, which can cause Q fever. Therefore, we aimed to develop a novel T cell-targeted vaccine that induces pathogen-specific cell-mediated immunity to protect against Q fever in humans while avoiding the reactogenicity of the current inactivated whole cell vaccine. Human HLA class II T cell epitopes from *C. burnetii* were previously identified and selected by immunoinformatic predictions of HLA binding, conservation in multiple *C. burnetii* isolates, and low potential for cross-reactivity with the human proteome or microbiome. Epitopes were selected for vaccine inclusion based on long-lived human T cell recall responses to corresponding peptides in individuals that had been naturally exposed to the bacterium during a 2007-2010 Q fever outbreak in the Netherlands. Multiple viral vector-based candidate vaccines were generated that express concatemers of selected epitope sequences arranged to minimize potential junctional neo-epitopes. The vaccine candidates caused no antigen-specific reactogenicity in a sensitized guinea pig model. A subset of the vaccine epitope peptides elicited antigenic recall responses in splenocytes from C57BL/6 mice previously infected with *C. burnetii.* However, immunogenicity of the vaccine candidates in C57BL/6 mice was dominated by a single epitope and this was insufficient to confer protection against an infection challenge, highlighting the limitations of assessing human-targeted vaccine candidates in murine models. The viral vector-based vaccine candidates induced antigen-specific T cell responses to a broader array of epitopes in cynomolgus macaques, establishing a foundation for future vaccine efficacy studies in this large animal model of *C. burnetii* infection.

## 1 Introduction

Q fever, a disease caused by the Gram-negative bacterium *Coxiella burnetii*, is highly infectious; a single inhaled organism can result in acute disease in humans, leading in ~5% of cases to chronic disease that can be severe (1–3). Zoonotic Q fever outbreaks have been documented in several countries including Australia and the Netherlands, and has been of concern to the United States (US) Department of Defense because of high seroconversion rates detected among military personnel serving in the Middle East (4–6). Doxycycline and other antibiotics can be used to treat acute Q fever, following clinical and serological confirmation of *C. burnetii* infection (2). However, a mild or asymptomatic infection may evade diagnosis, and can in some individuals subsequently progress to a persistent chronic infection, especially in those with specific risk factors such as advanced age or a history of cardiac valve surgery, or more commonly to post-infection Q fever fatigue syndrome (2, 7). A vaccine is thus considered critical to the prevention and control of Q fever in occupational and biodefense settings. Regulatory approval of Q-VAX^®^, an inactivated vaccine used in Australia, has been hindered in the US and Europe by reactogenicity in previously exposed individuals, and Q-VAX^®^ vaccination is contraindicated for individuals with a positive skin test for cell-mediated immune reactions to *C. burnetii* antigen or with serologically detected circulating anti-*Coxiella* antibodies (8–10). There is thus a need for an efficacious but less reactogenic vaccine for occupational and biodefense purposes.

Both humoral and cellular immune responses contribute to the control of *C. burnetii* infection and to the responses to whole cell vaccines (11–13). Results from studies in murine infection models suggest that T cell responses, particularly Th1 responses, are necessary for effective infection control (14–18), while antibodies alone are insufficient to clear infection (14, 17, 19). Vaccines comprising epitopes selected based on T cell responses observed in mice previously exposed to live or inactivated whole cell *C. burnetii* can reduce disease following infection challenge in mice (20, 21).

The objective of the Q-VaxCelerate program is to develop a non-reactogenic T cell-targeted vaccine that will protect against *C. burnetii* infection and disease in humans (22). A systematic process of computational epitope prediction and experimental validation, including screening for human T cell recall responses in individuals naturally exposed to the bacterium during the 2007-2010 Dutch Q fever outbreak, identified promiscuous human leukocyte antigen (HLA) class II epitope clusters from *C. burnetii* that are associated with long-lived T cell memory in humans (23). The corresponding peptide sequences represent candidates for inclusion in a vaccine that aims to elicit sustained T cell memory that can subsequently be recalled and boosted by natural exposure in an immunogenetically diverse human population.

Based on these previous epitope profiling data, we generated multiple viral vector-based vaccine candidates expressing epitope concatemers designed to establish anti-*C. burnetii* cellular immunity in humans. Vaccine development requires establishing immune response correlates in animal models that can inform selection of safe and effective vaccine doses for human testing (24, 25). To this end, we tested the vaccine candidates in three established animal models of immune responses to *C. burnetii*. The epitope concatemer vaccines did not elicit antigen-specific hypersensitivity responses in a guinea pig model of Q fever vaccine reactogenicity. While several of the vaccine epitopes stimulated antigenic recall responses in a mouse model of Q fever, vaccine immunogenicity was dominated by a single epitope in this model and this was insufficient to confer protection against *C. burnetii* challenge. However, broader immunogenic responses to the multi-epitope antigens were observed in cynomolgus macaques, suggesting that this nonhuman primate may be a more appropriate model for the evaluation of human-targeted T cell vaccines.

## 2 Materials And Methods

### 2.1 Bacteria

Axenic stock *C. burnetii* Nine Mile strain (*Cb*9M) was prepared using acidified citrate cysteine medium (ACCM-2) pH 4.75 (Sunrise Science Products, San Diego, CA, USA). Propagation was as described (26, 27), seeding cell free culture media with 1 x 10^6^ genome equivalents (GE) of *Cb*9M phase 1 RSA 411 (BEI Resources, Manassas, VA, USA). Flasks were incubated for nine days on a shaker (75 rpm) at 37°C in a 2.5% O_2_, 5% CO_2_ environment. Bacteria were recovered by centrifugation at 14,000 x g for 30 min, resuspended in sucrose phosphate buffer (28) and stored at -80°C. The stock aliquots were found to contain 2.17 x 10^7^ GE per µL as determined by qPCR (see below). Infectivity of stock was confirmed previously by passage through mice (A/JCr) (27).

#### 2.1.1 Quantitative PCR (qPCR)

Genomic DNA was extracted from samples using standard techniques (QIAamp DNA mini blood kit, Qiagen, Valencia, CA, USA) and qPCR was performed to detect *C. burnetii* targets using LSI VetMax™ *Coxiella burnetii* Absolute Quantification kit (Life Technologies, Lissieu, France).

### 2.2 Reference Vaccine

The veterinary vaccine, Coxevac^®^ was obtained from CEVA Sante Animale (Lot 0101Eg1A, Libourne, France). This formalin inactivated phase I *C. burnetii* corpuscular antigen formulation is preserved with thiomersal and marketed for annual use in ruminants. Dosing in this study was based upon the assumption that Coxevac^®^ was standardized at 100 µg of antigen per mL (29). The vaccine was determined to contain 86 µg of protein per mL (BCA protein assay, Pierce Biotechnology, Rockford, IL, USA) and qPCR indicated that the vaccine contained 1.3x10^8^ GE per mL.

### 2.3 Experimental Animals and Ethics Statement

#### 2.3.1 Ethics Statement

Mice for immunogenicity studies were used in accordance with the UK Animals (Scientific Procedures) Act 1986 under project license number 30/3385 granted by the UK Home Office; the experimental protocols were approved by the Oxford University Animal Welfare and Ethical Review Committee (AWERB). Animal research protocols for murine vaccine challenge studies were reviewed and approved by the Colorado State University (CSU) Institutional Animal Care and Use Committee (IACUC) (16–6,844). Animal research protocols for guinea pig experiments were also reviewed and approved by the CSU IACUC (14-5305A, 16-6844A). The animal research protocol for vaccine immunogenicity assessment in cynomolgus macaques was reviewed and approved by the Massachusetts General Hospital IACUC (2019N000076). All animal experimental activities were conducted in full compliance with the applicable institutional, federal and international regulations, and protocols were further reviewed and approved by the US Defense Threat Reduction Agency’s Animal Care and Use Review Office.

#### 2.3.2 Mice

For vaccine immunogenicity and dosing studies, 6- to 8-week old female C57BL/6 mice were obtained from Harlan (UK). Mice were housed in specific pathogen-free conditions.

For studies involving *C. burnetii*-infected mice, female wild-type C57BL/6 mice (6–8 weeks old) were obtained from Charles River Laboratories (Wilmington, MA, USA). Mice were maintained under BSL3 conditions in microisolator cages (Smart Flow, Tecniplast, Westchester, PA, USA) at the Regional Biocontainment Laboratory, Colorado State University, Fort Collins, CO, USA. Animals were provided water and rodent chow *ad libitum* and evaluated daily to detect changes in body weight, body condition, behavior, and activity level.

#### 2.3.3 Guinea Pigs

Female Dunkin-Hartley Crl:HA guinea pigs (300 g/35-42 days of age) were purchased from Charles River Laboratories (Wilmington, MA, USA). All guinea pigs were maintained under biosafety level (BSL) 3 conditions in isolator cages (Smart Flow, Techniplast, Westchester, PA, USA) at the Regional Biocontainment Laboratory at Colorado State University (Fort Collins, Colorado, USA). Animals were provided water and guinea pig chow *ad libitum*. Dried fruit and hay pellets were offered for enrichment. Clinical evaluations were made daily on each animal throughout the study period to detect changes in body weight, body condition, behavior and activity level. Body temperature was documented at four hour intervals by temperature recorders (Thermochron iButton^®^, Maxim Integrated Products, Inc., Sunnyvale, CA, USA) implanted in the abdominal cavity *via* flank incision.

#### 2.3.4 Cynomolgus Macaques

Male cynomolgus macaques (4-7 kg) were purchased from Charles River Laboratories (Wilmington, MA, USA). All macaques were housed in social pairs in modulated stainless steel caging equipped with a perch or resting surface and providing sufficient space for physical activity. Animals were provided water ad libitum, with standard Purina Lab Monkey Diet and ¼ piece of fresh produce provided daily. Manipulanda supplied included standard rubber, plastic, and stainless steel enrichments as well as novel toys and enrichment creations distributed weekly. Animals were assessed for abnormal or undesirable behaviors in twice-weekly behavioral rounds. Following vaccine administration, animals were monitored daily for signs of discomfort, and for signs of irritation or inflammation at the site of vaccine injection.

### 2.4 Assessment of Class II Epitope Antigenicity in C57BL/6 Mice

C57BL/6 mice (n=3 per group) were inoculated intranasally with 1x10^5^ GE of *C. burnetii* Nine Mile or with saline control. The mice were evaluated clinically from day 7 to termination with body weight and clinical score recorded daily. Mice were euthanized at day 51 post-infection and splenocytes recovered for ELISpot analysis.

#### 2.4.1 *Ex Vivo* ELISpot Assay of Splenocytes from *C. burnetii*-Infected Mice

Isolation and assay of splenocytes from *C. burnetii-*infected mice were carried out under BSL3 containment. The frequency of epitope-specific splenocytes was determined by IFNγ ELISpot assay using the colorimetric Mabtech IFNγ ELISpot Kit with pre-coated plates according to the manufacturer’s protocol. Washed splenocytes in RPMI 1640 (Gibco) supplemented with 10% fetal calf serum (FCS, Atlanta Biologicals) were added at 2.5x10^5^ cells per well. Assays included 4 μg/mL anti-CD28 (BD Pharmingen, USA) and 4 μg/ml anti-CD137 (BioCell, USA) as co-stimulants. Individual peptides were added at 2 µg/mL in triplicate wells. Triplicate wells were stimulated with 2 μg/mL Concanavalin A (ConA; Sigma Aldrich) as a positive control for cell viability, and six replicate wells with medium containing 1% DMSO were used for background determination. Triplicate wells stimulated with 9 μg/mL purified recombinant Com1 protein (gift from Dr. Wei-Mei Ching, Viral and Rickettsial Diseases Department, US Naval Medical Research Center, Silver Spring, MD, USA) or with 2 μg/mL of Coxevac® as positive controls for *C. burnetii*-specific antigenic recall responses (the amount of Coxevac® included in the assays was limited by the concentration of the commercial formulation). Raw spot counts were recorded by photographic imaging of ELISpot plates under an OMAX microscope within the BSL3 facility. ELISpot well images were transferred electronically and raw spot counts were analyzed using the AID ELISpot software v7.0 (AID Diagnostika GmbH). Results were calculated as the average number of spots in the peptide wells minus the average number of spots in the background determination wells, adjusted to spots per one million cells. High spot density in the ConA-stimulated wells resulted in lack of separation between individual spots that precluded accurate counting by either the analysis software or manual counting; based on approximate manual counts, these wells averaged >800 spot-forming units per one million cells.

### 2.5 Vaccine Computational Design

String-of-epitopes constructs were designed by concatenating select epitopes head-to-tail in random order. To avoid production of neo-epitopes at epitope junctions, the VaxCAD algorithm was applied to arrange epitopes in an order that diminishes potential junctional immunogenicity (30). The final epitope arrangements did not require insertion of spacers between epitopes to disrupt potential junctional immunogenicity unresolved by VaxCAD. The potential for transmembrane insertion was assessed using TMHMM v2.0 [https://services.healthtech.dtu.dk/service.php?TMHMM-2.0 (31)]. No transmembrane stretches necessitating epitope rearrangement were predicted. Concatemer-encoding nucleic acid sequences, including an N-terminal tissue plasminogen activator (TPA) signal sequence and C-terminal V5 expression tag and codon-optimization for expression in human cells, presented no obvious major concerns when analyzed for RNA secondary structure using the RNAfold WebServer [http://rna.tbi.univie.ac.at/cgi-bin/RNAWebSuite/RNAfold.cgi (32)] and the RNAstructure Fold Web Server [http://rna.urmc.rochester.edu/RNAstructureWeb/Servers/Fold/Fold.html (33)].

Epitope concatemer-protein fusion designs utilized protein sequences for *C. burnetii* Com1 (UniProt ID: H7C7D7) or *Mycobacterium tuberculosis* HSP70 (UniProt ID: P9WMJ9).

### 2.6 Vaccine Construct Generation and Production

An E1- and E3-deficient human adenovirus serotype 5, the ChAdOx2 derivative of the chimpanzee adenovirus (ChAd) 68 (34), and Modified vaccinia virus-Ankara (MVA (35) were used as vaccine vectors. DNA sequences corresponding to vaccine antigen designs were synthesized and cloned into plasmids containing *att*R1 and *att*R2 recombination sites (Gateway^®^ Life technologies, CA, USA) under control of a CMV promotor. Antigen-encoding inserts were then transferred into the vaccine vectors by recombination as previously described (36–38). The adenoviral vaccine candidates were produced as previously described using the HEK293A cell line (Invitrogen), which contains a stably integrated copy of the E1 gene that supplies the E1 proteins (E1a and E1b) required to generate recombinant adenovirus (39), by the Viral Vector Core Facility of the Jenner Institute, University of Oxford, UK. MVA vaccines were produced using the DF-1 cell line and purified using sucrose cushion centrifugation (40). Virally vectored vaccines were formulated in endotoxin-free PBS. All constructs were verified by sequencing. Vaccine constructs generated are summarized in **Table 1** and described further in the *Results*.

**Table 1:** Summary of epitope concatemer vaccine constructs.

Vaccine Insert Design	Insert Name	Vaccine Vector		
		HuAd5	ChAdOx2	MVA
TPA/18-tope/V5	QVx18	Yes	Yes	not made
TPA/27-tope/V5	QVx27	Yes	Yes	not made
Kozak/TPA/18-tope/V5	kQVx18	Yes	Yes	not made
Kozak/TPA/27-tope/V5	kQVx27	Yes	Yes	Yes
TPA/18-tope/Com1/V5	QVx18-Com1	Yes	Yes	not made
TPA/MtbHSP70/18-tope/V5	MtbHSP70-QVx18	Yes	Yes	not made

### 2.7 Assessment of Vaccine Insert Expression

#### 2.7.1 Western Blot Analysis of Epitope Concatemer Expression

Plasmid clones carrying verified antigen-encoding inserts were transfected into HEK293 cells (2.5 µg of plasmid DNA). Controls underwent a mock transfection. Cells were harvested at 24, 48, or 72 hr after transfection, and proteins in cell lysates were separated by polyacrylamide gel electrophoresis (NU-Page pre-cast BIS-TRIS 4-12% gradient gels). Separated proteins were transferred to PVDF membranes. Blots were probed with anti-V5 antibody (rabbit IgG Poly29038, Biolegend catalog #903802). Bound anti-V5 antibody was detected using an anti-rabbit secondary antibody (mouse IgG anti-rabbit-HRP, Rockland catalog #18-8816-31) visualized using the Pierce ECL system (catalog #32106).

#### 2.7.2 Flow Cytometry Analysis

HeLa cells were infected with adenoviruses expressing transgenes at a MOI of 100. Transfected and infected HeLa cells were left overnight at 37°C with 5% CO_2_. Vaccine antigen expression was assessed by flow cytometry using FITC-conjugated rabbit anti-V5 Tag antibody (#A190-120A, Bethyl Laboratories).

### 2.8 Assessment of Vaccine Immunogenicity in C57BL/6 Mice

#### 2.8.1 Vaccinations

Mice received HuAd5 vaccines (10^9^ infectious units, IU) and MVA-kQVx27 boost vaccinations (10^7^ IU) as indicated; all vaccines were delivered intramuscularly (IM) in saline. Each dose was delivered as 50 µL in each hind leg (100 µL total). Splenocytes were harvested 21 days (HuAd5 vaccines) or 7 days (MVA-kQVx27 boost) after the final vaccination. Briefly, spleens were mashed and filtered into a single cell suspension and red blood cells lysed in ACK lysis buffer (0.15 M NH_4_Cl, 1 mM KHCO_3_, 0.1 M Na_2_EDTA, pH ∼ 7.3) before resuspension in complete α-MEM containing 10% FCS, 2 mM l-glutamine, 0.1% β-mercaptoethanol, 100 U penicillin and 100 μg streptomycin.

#### 2.8.2 *Ex Vivo* IFNγ ELISpot Assay of Epitope-Specific Responses to Vaccine Constructs

A Mouse IFN-γ FluoroSpot kit (Mabtech) was used to measure antigen-specific T cell responses in mouse spleen tissue. Anti-IFN-γ monoclonal capture antibodies were diluted in PBS to a concentration of 15 μg/ml. The IPFL plate membrane was washed with 15 μL of 35% ethanol per well for no more than 60 s and then washed five times with 200 μL of sterile H_2_O per well. 100 μL of capture antibody was added to each well and the plate was sealed for overnight incubation at 4°C. The plate was washed five times with 200 μL of sterile PBS per well the following day and the wells were then blocked with DMEM containing 10% FBS for 30 mins at room temperature. Stimuli, either DMSO (1:100) as a negative control, ConA (12 μg/mL) as a positive control, individual peptides corresponding to vaccine epitopes (2 µg/mL), or a Com1 peptide pool (6 µg/mL) composed of a series of overlapping peptides covering the full-length Com1 protein sequence (15mers overlapping by 11 amino acids; manufactured by thinkpeptides, ProImmune), were added to the appropriate wells, followed by 2 x 10^5^ splenocytes from the appropriate sample to each corresponding well. The plate was sealed and incubated overnight at 37°C in a 5% CO_2_ incubator. Cells were removed from the wells the following day and the plate was washed five times with 200 μL of PBS per well. Anti-IFN-γ-R4-6A2-BAM detection antibody was diluted in PBS containing 0.1% BSA to a concentration of 1:200. 100 μL of detection antibody mixture was added to each well and incubated for two hours at room temperature. Anti-BAM-490 fluorophore conjugate was diluted to a concentration of 1:200 with PBS containing 0.1% BSA and, after washing the plate five times with PBS, 100 μL of this dilution were added to each well. The plate was wrapped in aluminium foil and incubated for one hour in the dark at room temperature. The plate was washed five times with PBS before adding 50 μL of fluorescence enhancer to each well and incubating in the same manner for 15 mins. The plate was emptied of all liquid and the underdrain was removed. Plates were completely dried in the dark at room temperature prior to spot counting with AID ELISpot software 8.0 (Autoimmune Diagnostika). Excitation 490 nm/emission 510 nm (FITC) wavelengths were used to measure IFN-γ.

### 2.9 Vaccine Efficacy Study in C57BL/6 Mice

Sixty C57BL/6 mice (6-8 weeks old at study initiation) were randomly assigned to 6 groups of 10 animals each, and vaccinated as indicated in **Table 2**. At study day 140 (16 days after the second boost), mice were chemically restrained (intraperitoneal ketamine, 100 mg/kg, and xylazine, 10 mg/kg) for intranasal inoculation with 2x10^3^ GE (as determined by qPCR) of *C. burnetii* in 20 µL. Mice were euthanized at day 154 (two weeks after challenge). Spleens were harvested and weighed, and spleen-to-body weight ratio determined. Splenocytes were collected and the spleen bacterial burden was determined from homogenates by qPCR.

**Table 2:** Vaccination groups and schedule for C57BL/6 vaccine efficacy study.

Group	N	Day 0 (prime vaccination)	Day 103 and 124 (boost vaccinations)
Unvaccinated	10	Saline 100 µL IM	Saline
Positive Control	10	Coxevac 10 µg SC	None
Vaccinated	10	1x10^9^ IU HuAd5-QVx18	1x10^7^ IU MVA-kQVx27
Vaccinated	10	1x10^9^ IU HuAd5-QVx18-Com1	1x10^7^ IU MVA-kQVx27
Vaccinated	10	1x10^9^ IU HuAd5-QVx27	1x10^7^ IU MVA-kQVx27
Vector Control	10	1x10^9^ IU HuAd5 Empty vector	1x10^7^ IU MVA-GFP

Virally-vectored vaccines were administered IM; each dose was delivered as 50 µL in each hind leg (100 µL total). Animals were challenged on study day 140 (16 days after the second boost) by intranasal exposure to C. burnetii.

### 2.10 Assessment of Vaccine Reactogenicity in Guinea Pigs

Female guinea pigs were sensitized by intranasal inoculation with 10^6^ GE of *C. burnetii* Nine Mile strain or saline in 100 µL volume, as described previously (41). Blood samples were collected from chemically restrained animals by venipuncture at day 42 post-sensitization for serum collection and evaluation of serological status. Reactogenicity of vaccine candidates relative to positive and negative controls was evaluated by intradermal (i.d.) challenge at day 42 post-infection (p.i) (**Table 3**). Challenge with 1 µg Coxevac^®^ whole cell vaccine (Ceva Sante Animale, Libourne, France) was used as a positive control; negative controls consisted of saline injections. Gross reactions were monitored at 3 hr and 8 hr post-challenge and daily thereafter. On day 7 post-challenge, animals were anesthetized (ketamine 40 mg/kg and xylazine 5 mg/kg, i.p.) and euthanized with Beuthanasia (i.p.). Inoculation sites were excised from euthanized animals by punch biopsy (12mm AcuPunch, Acuderm Inc., Ft. Lauderdale, FL, USA) and collected in 10% formalin for paraffin embedding and stained with hematoxylin and eosin for histopathological review. Histological reactions at injection sites were scored by an experimenter blinded to the treatment group, using the criteria previously described (41). Briefly, a score of 0 indicates no inflammation, 1 indicates localized macrophage dominated inflammation, 2 macrophage dominated inflammation with limited tissue infiltrations, 3 lymphocytic inflammatory infiltrates extending into the deep dermis, 4 edema and increased pyogranulomatous inflammation extending deep into the subcutis, and 5 widespread pyogranulomatous inflammation including necrosis.

**Table 3:** Challenge inoculations for reactogenicity testing in guinea pigs.

Vaccine Candidate	Dose
PBS negative control	100 µL
Coxevac^®^ positive control	1 µg *C. burnetii* antigen
ChAdOx2-QVx18	1.78x10^8^ IU
ChÁdOx2-QVx27	1.54x10^8^ IU
ChAdOx2-QVx18-Com1	1.4x10^8^ IU
ChAdOx Empty	1.0x10^8^ IU
MVA-kQVx27	1.3x10^7^ IU
MVA Empty	1.6x10^7^ IU

All challenge inoculations were administered in 100 µL volume. IU, infectious units.

#### 2.10.1 Serology

Serological status of sensitized guinea pigs was determined by ELISA using the Q Fever antibody test kit (IDEXX Laboratories Inc., Westbrook, ME, USA) with the secondary antibody replaced with peroxidase conjugated protein A/G at 1:10,000 dilution (EMD Millipore Corp., Billerica, MA, USA); the kit substrate is 3,3’,5,5’-tetramethylbenzidine (TMB). Results are presented as optical density at 450 nm (OD_450_). Day 0 samples were tested at the kit recommended dilution of 1:400 and D42 samples were tested at 1:1000.

### 2.11 Assessment of Vaccine Immunogenicity in Cynomolgus Macaques

#### 2.11.1 Vaccinations and Sample Collection

Four male Mauritian cynomolgus macaques (4-7 kg) were assigned to two vaccine groups (n=2 per group). On the day of vaccination, the animals were sedated (0.015 mg/kg dexmedetomidine hydrochloride plus 2-4 mg/kg ketamine) and vaccinated intramuscularly on the arm with 500 μl of vaccine in isotonic phosphate-buffered saline (PBS). Animals received either ChAdOx2-QVx18-Com1 or ChAdOx2-QVx27 (each formulated at 2x10^6^ IU/μl) as prime vaccination. Heterologous boost vaccinations, administered 8 and 11 weeks after the prime vaccination, were performed using MVA-kQVx27 (formulated at 2x10^5^ IU/μl). The timing of blood draws and spacing of vaccinations were based on established University of Oxford protocols for these vaccine platforms (42). After each vaccination, the animals were monitored for 15 minutes for any local or systemic reaction to the vaccine, then released from sedation by administration of 0.3 mg/kg atipamezole hydrochloride.

Whole blood (up to 50 mL) was drawn into K_2_EDTA vacutainer tubes from peripheral veins prior to vaccination and at 2, 4, 8, 9, 11, 12 and 14 weeks after the prime vaccination. Peripheral blood mononuclear cells (PBMC) were isolated from these samples using a 60% percoll gradient. In brief, whole blood was diluted with PBS at a ratio of 1:2. The diluted whole blood was layered on top of 60% percoll at a ratio of 1:1. The sample was centrifuged at 800 g for 30 min at 15°C with no brakes applied during deceleration. The buffy coat that was collected after centrifugation was washed in PBS, and thereafter erythrocytes lysed with red blood cell lysis buffer. Post-lysis the cells were washed and resuspended in RPMI medium supplemented with 10% fetal bovine serum, 1% L-glutamine, 100 international units/mL penicillin, and 100 µg mL streptomycin. For each stimulation condition, one million cells were plated per well in a 96-well round bottom plate, and rested for 2 hours at 37°C with 5% CO_2_.

#### 2.11.2 *Ex Vivo* Measurement of Immunogenic T Cell Responses

Plated PBMCs from individual animals were cultured at 37°C with 5% CO_2_ for 25 hr. in the presence of one of 7 vaccine epitope peptide pools, a pool of peptides representing the full Com1 protein sequence, a peptide concatemer of non-*Coxiella* sequences, or no peptide stimulation. Vaccine epitope peptide pools comprised the following sets (epitopes are as defined in **Table 4**): Pool A (p14, p15, p20, p21); Pool B (p18, p22, p37, p38); Pool C (p04, p12, p17, p26); Pool D (p27, p30, p42, p43); Pool E (*p02*; p45; p48); Pool F (*p19, p23, p46, p47*); Pool G (*p06, p24, p31, p50*); epitopes in *italics* are included in QVx27 but not QVx18 constructs, though *p02* is derived from Com1 and this epitope is thus present in the QVx18-Com1 fusion antigen. The final peptide concentration in the stimulation cultures with the *Coxiella* peptide pools was 2 µg/mL of each vaccine epitope peptide. The final concentration of the Com1 peptide pool was 6 µg/mL. The non-*Coxiella* peptide concatemer (Biotin-[PEG4]-LEQLERVKR–**VSGLEQLESIINFEKLTEWTS**-RVKR-*EKFDPLGQLSIFYHKTY*-amide) contained two control sequences: a sequence from ovalbumin (**bold**) encompassing the immunodominant epitope SIINFEKL and a scrambled peptide sequence (*italics*) with no significant homology to mammalian proteins. The concentration of the non-*Coxiella* peptide concatemer in the stimulation cultures was 1 µg/mL. The Com1 peptide pool was included as a stimulation condition from week 4 post-prime vaccination, and the non-*Coxiella* epitope concatemer from week 8 after the prime vaccination. Brefeldin A (5 µg/mL) was added for the final 5 hours. Cell viability in all PBMC preparations was confirmed by responses to a 5 hr. stimulation with phorbol-12-myristate 13-acetate (40.5 nM) and ionomycin (669.3 nM) in the presence of Brefeldin A (5 µg.mL).

**Table 4:** HLA class II epitopes selected for vaccine design.

ID	Epitope	Source antigen			% donors responding	
		CBU Code	UniProt ID	Gene Name	All IGRA+	IGRA+ with prior symptomatic Q fever
*p2*	TPTFVIGNKALTKFGF	CBU_1910	H7C7D7	com1	16	26
p4	KIGVIKAIRTITGLGLKEA	CBU_0229	P0C8S3	rplL	23	30
*p6*	SHEVLHAMSRGVEVLA	CBU_1718	P19421	groL	7	17
p12	GKHFDGIKVLKLSPQNTI	CBU_1869	Q83AL4		21	22
p14	PDYVLNAVNHIRYKP	CBU_1835	Q83AP6	protoporphyrinogen oxidase	25	30
*p15	MMEHLQNITNLVSTGRQGA	CBU_1835	Q83AP6	protoporphyrinogen oxidase	20	30
p17	KIPVKIIKPPFVRRG	CBU_1716	Q83B06	gcvT	28	30
*p18	QGHIINIGSISSHQV	CBU_1513	Q83BJ5	short chain dehydrogenase	14	17
*p19*	EAVYKGFTPLKAEDIAEA	CBU_1513	Q83BJ5	short chain dehydrogenase	20	26
*p20	AQPIIHRLSTGQNTNP	CBU_1416	Q83BT6	repressor protein C2	9	4
*p21	IARYFMVNISQLIGEE	CBU_1416	Q83BT6	repressor protein C2	14	22
p22	RLGFMSFFTKAVVEALKRF	CBU_1398	Q83BU7	sucB	23	22
*p23*	REAVLFLVTIKELLEDP	CBU_1398	Q83BU7	sucB	18	26
*p24*	LPPVTSSVAVKVPSS	CBU_1260	Q83C69	OmpA-like transmembrane domain protein	21	26
*p26	QTQLQQSFSKRTMAT	CBU_1221	Q83CA7	membrane-spanning protein	7	4
*p27	RFDLSLMLNYPNSADRY	CBU_1157	Q83CG1		12	17
*p30	GTEITVQKASIASVLPK	CBU_1143	Q83CH2	yajC	11	9
*p31*	AENVLIIHNKTLAHRYLA	CBU_0968	Q83CY3	phospholipase D	11	4
*p37	VAKLRGDLSSIIHKL	CBU_0718	Q83DK8	membrane-associated protein	18	26
*p38	LSSIIHKLTSFSKTEA	CBU_0718	Q83DK8	membrane-associated protein	21	26
*p42	DHAYKLAVSSTKSMT	CBU_0497	Q83E37	fabF	5	0
*p43	NAGIIRNKLKIQATIN	CBU_0383	Q83EE1	tag	5	0
*p45	GVAYTYNRANAGLPTNK	CBU_0307	Q83EL2	outer membrane protein	13	9
*p46*	VPGYRNASSKRFVAP	CBU_0307	Q83EL2	outer membrane protein	20	17
*p47*	KAQLIQLKTHVTINAT	CBU_0109	Q83F42	methionine-binding protein	7	13
p48	SPAVLSAAKKIFGDGA	CBU_0109	Q83F42	methionine-binding protein	23	30
*p50*	LRPVRYFTGVPSPVKTPE	CBU_1200	Q9ZH99	icd	20	22

Epitope IDs are those reported by Scholzen et al. (23). Recall responses to epitopes were previously assessed by cultured IFNγ ELISpot in individuals with demonstrated responses to heat-killed C. burnetii in a whole blood IFNγ release assay (IGRA) (23). The nine epitope IDs in italics indicate those included only in the 27-epitope vaccine designs. All other epitopes were included in all vaccine designs. * = antigenic recall responses observed in Coxiella-infected C57BL/6 mice.

T cell activation status following peptide stimulation was evaluated by flow cytometry. Stimulated cells were washed in PBS, and thereafter were incubated with 1:250 dilution of Zombie UV fixable viability dye (Biolegend) for 30 minutes at room temperature. Cells were washed and resuspended with cell staining buffer (PBS containing 1 mg/mL bovine serum albumin and 2 mM Na_2_EDTA), then incubated for 30 minutes with antibodies against cell surface antigens: CD3 (SP34-2, BUV395, BD Biosciences), CD4 (OKT4, BV510, Biolegend), CD8 (SK1, APC Fire750, Biolegend), CD11c (3.9, BV785, Biolegend), CD20 (2H7, PerCP-Cy5.5, Biolegend), CD16 (3G8, PerCP-Cy5.5, Biolegend), CD14 (M5E2, PerCP-Cy5.5, Biolegend) and CD66b (TET2, PerCP-Vio770, Miltenyi Biotec). Surface-stained cells were then washed, fixed and permeabilized using isotonic fixation/permeabilization buffer (eBioscience). After this, cells were incubated for 30 minutes with antibodies against intracellular antigens: CD154 (24-31, APC, Biolegend), IFNγ (B27, PE, Biolegend), IL-2 (MQ1-17H12, BV421, Biolegend), Perforin (Pf-344, FITC, Mabtech), TNFα (Mab11, BV711, Biolegend) and T-bet (4B10, PE-Cy7, Biolegend). Fully stained cells were washed and treated with 1.6% paraformaldehyde for 10 minutes. The fully fixed cells were washed and resuspended in cell staining buffer, then stored overnight at 4°C. Data were acquired the next day on a BD LSRFortessa X-20 and analysed using FlowJo™ Software (BD Life Sciences).

### 2.12 Statistical Analysis

Statistical analyses were performed using GraphPad Prism v9 (San Diego, CA, US).

## 3 Results

### 3.1 Generation of Multi-Epitope T Cell-Targeted Vaccines

#### 3.1.1 Epitope Selection

To support rational selection of epitopes for inclusion in a vaccine for prevention of Q fever disease in humans, previous work identified 44 promiscuous HLA class II epitope clusters from *C. burnetii* that were associated with long-lived human T cell recall responses in individuals naturally exposed to the bacterium during a 2007-2010 Q fever outbreak in the Netherlands (23). The identified epitope clusters are derived from multiple antigens, are conserved in seven publicly available *C. burnetii* genome sequences, and cover eight human HLA-DR supertype alleles. A subset of these epitope clusters, including 23 epitopes that elicited recall responses in at least 10% of individuals with known prior exposure to *C. burnetii* or previous symptomatic Q fever, were selected for inclusion in multi-epitope concatemers as vaccine candidates (**Table 4**).

**Figure 1:**
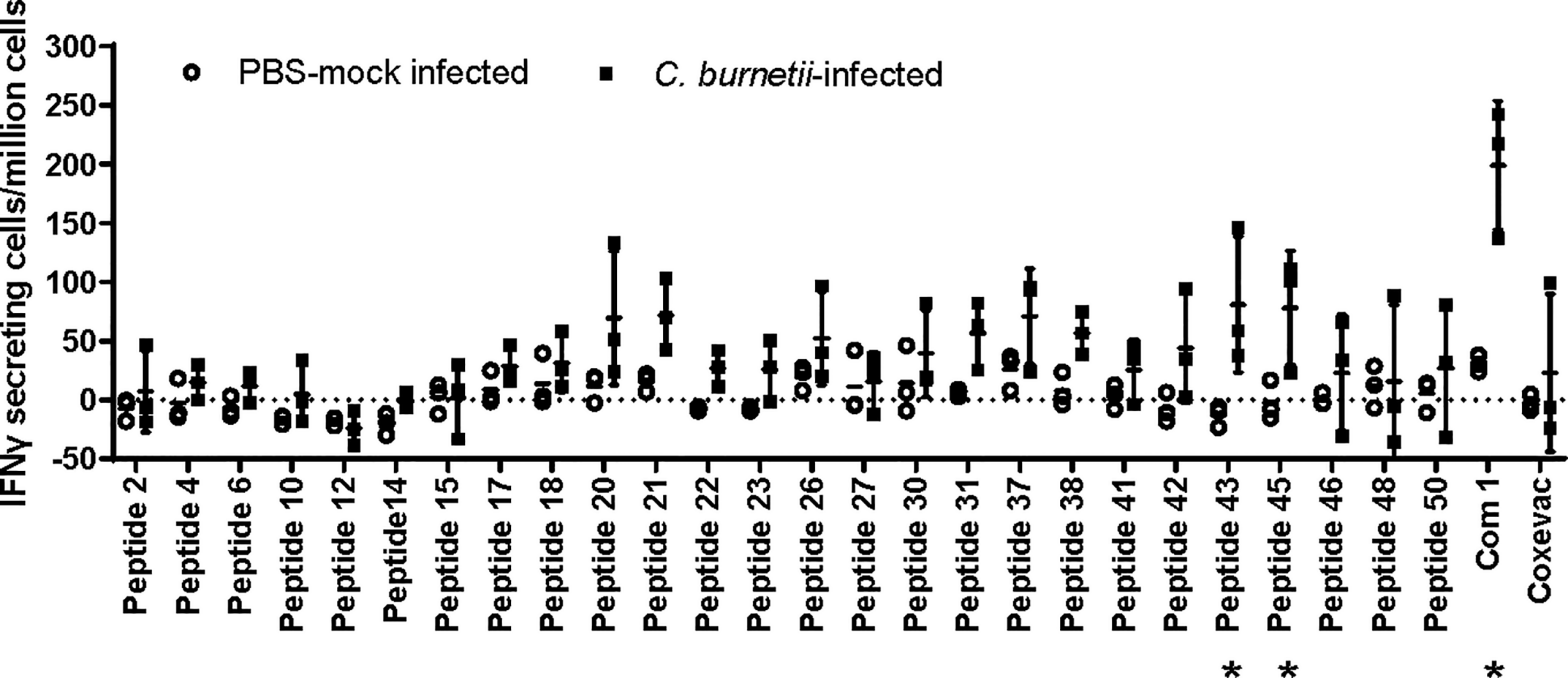
Antigenic recall responses in *Coxiella*-infected C57BL/6 mice. C57BL/6 mice (n=3 per group) were infected intranasally with 1x10^5^ GE of *C. burnetii* Nine Mile strain, or mock-infected with PBS. Mice were terminated at day 51 post-infection and splenocytes recovered for ELISpot analysis. Splenocytes were stimulated with individual peptides, recombinant Com1, or the veterinary vaccine Coxevac in the presence of costimulants anti-CD28 and anti-CD173. Data are presented as IFNγ-secreting cells (spot-forming units)/million splenocytes; bars indicate group mean and standard deviation. * = p <0.05 for differences between mock-infected and *C. burnetii*-infected animals by two-way ANOVA with Sidak’s multiple comparison test.

In anticipation of testing vaccines comprising these human antigenic HLA class II epitopes in a C57BL/6 mouse *C. burnetii* infection model, we assessed C57BL/6 post-infection antigenic recall responses to 26 epitopes associated with observed recall responses in humans and predicted binding potential for the MHC class II I-Ab allele of C57BL/6 mice [assessed using the EpiMatrix algorithm (30)]. Two of the epitopes (p43, p45) as well as recombinant Com1 protein consistently stimulated IFNγ T-cell recall responses in splenocytes from *C. burnetii*-infected mice, with additional peptides inducing elevated recall responses in some individual mice (**Figure 1**). The veterinary vaccine Coxevac induced variable *ex vivo* recall responses despite containing whole cell antigens, perhaps due to interference with *ex vivo* cellular antigen processing and presentation by the formalin inactivation used in Coxevac production (43) or to constraints on the volume of commercial formulation that could be included in the assay. To expand the potential for vaccine responses in the C57BL/6 model, four additional epitopes (p20, p26, p42, and p43) were selected based on these murine antigenic recall responses for inclusion in vaccine designs, despite eliciting recall responses in <10% of the *C. burnetii*-exposed humans evaluated in the previous human antigenicity screening (**Table 4**).

#### 3.1.2 Multi-Epitope Antigen Design

**Figure 2:**
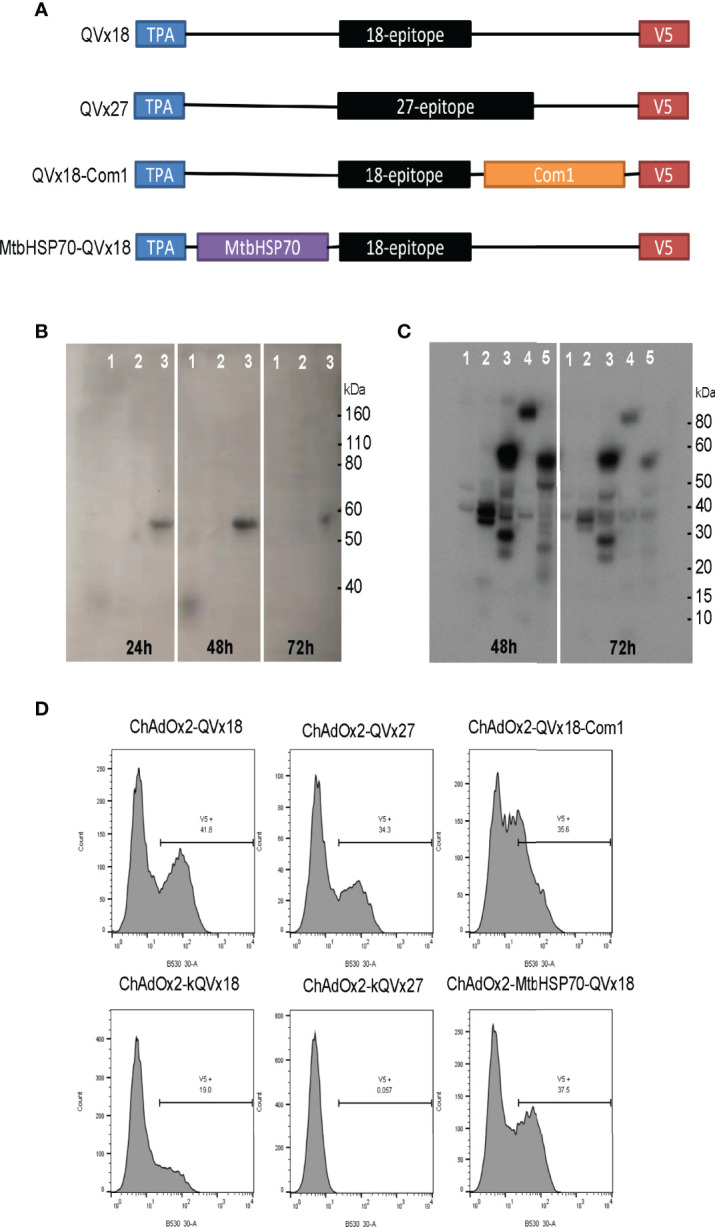
Design and expression of multi-epitope vaccine antigens. **(A)** Vaccine antigen designs. Sequences of 18-epitope and 27-epitope concatemers are given in **Table 5**. **(B, C)** Western blot analysis of epitope concatemer expression in HEK293 cells transfected with plasmids encoding the vaccine antigen inserts. Expected molecular weights: QVx18 concatemer, 36 kDa; QVx27 concatemer; 54 kDa; QVx18-Com1, 59 kDa; MtbHSP70-QVx18, 102 kDa. Vaccine antigen designs evaluated in **(B)** 1: QVx18; 2: empty plasmid; 3: QVx27. Vaccine antigen designs evaluated in **(C)** 1: empty plasmid; 2: kQVx18; 3: QVx18-Com1; 4: MtbHSP70-QVx18; 5: kQVx27. **(D)** Expression of vaccine antigens in HeLa cells infected with ChAdOx2 vaccine constructs detected by anti-V5 flow cytometry.

Four multi-epitope antigen designs were developed that incorporate the selected human immunoreactive peptide sequences (**Figure 2A**). Epitope concatemers were designed from two epitope sets: (a) all 27 selected epitopes (concatemer QVx27) and (b) an 18-epitope subset (QVx18) comprising 14 epitopes associated with particularly strong recall responses (stimulated:unstimulated response ratio >3) in at least 10% of *C. burnetii*-exposed humans (23) plus the four additional epitopes selected based on antigenic recall responses in *C. burnetii*-infected mice. Each epitope set was randomly concatenated and analyzed for non-specific potential immunogenicity at epitope junctions as well as the potential for transmembrane insertion, and rearrangements were made to minimize the potential of both. Immunogenicity at epitope junctions was assessed for mouse (C57BL/6) and human supertype HLA class II alleles to generate antigen designs with pre-clinical to clinical translation potential. The final epitope arrangements have no predicted immunogenicity at the epitope junctions nor potential for transmembrane insertion (**Figures S1, S2**). Final antigen designs for each concatemer incorporated a C-terminal V5 epitope tag for expression monitoring and N-terminal tissue plasminogen activator (TPA) signal sequence for concatemer secretion and uptake by the exogenous antigen processing pathway (**Table 5**).

**Table 5:** Epitope concatemer vaccine designs.

Concatemer	Amino Acid Sequence
***TPA***/18-tope/***V5*** (QVx18)	***MDAMKRGLCCVLLLCGAVFVSPS*** IARYFMVNISQLIGEESPAVLSAAKKIFGDGAAQPIIHRLSTGQNTNPRFDLSLMLNYPNSADRYQTQLQQSFSKRTMATMMEHLQNITNLVSTGRQGAKIGVIKAIRTITGLGLKEAKIPVKIIKPPFVRRGGKHFDGIKVLKLSPQNTIGVAYTYNRANAGLPTNKLSSIIHKLTSFSKTEAVAKLRGDLSSIIHKLPDYVLNAVNHIRYKPGTEITVQKASIASVLPKQGHIINIGSISSHQVNAGIIRNKLKIQATINDHAYKLAVSSTKSMTRLGFMSFFTKAVVEALKRF ***GKPIPNPLLGLDST***
***TPA***/27-tope/***V5*** (QVx27)	***MDAMKRGLCCVLLLCGAVFVSPS*** IARYFMVNISQLIGEESPAVLSAAKKIFGDGAAQPIIHRLSTGQNTNPRFDLSLMLNYPNSADRYQTQLQQSFSKRTMATMMEHLQNITNLVSTGRQGAKIGVIKAIRTITGLGLKEAKIPVKIIKPPFVRRGGKHFDGIKVLKLSPQNTITPTFVIGNKALTKFGFSHEVLHAMSRGVEVLAVPGYRNASSKRFVAPEAVYKGFTPLKAEDIAEALRPVRYFTGVPSPVKTPELSSIIHKLTSFSKTEAVAKLRGDLSSIIHKLPDYVLNAVNHIRYKPGVAYTYNRANAGLPTNKKAQLIQLKTHVTINATAENVLIIHNKTLAHRYLAGTEITVQKASIASVLPKREAVLFLVTIKELLEDPLPPVTSSVAVKVPSSQGHIINIGSISSHQVNAGIIRNKLKIQATINDHAYKLAVSSTKSMTRLGFMSFFTKAVVEALKRF ***GKPIPNPLLGLDST***

**N-terminal TPA signal sequence**. Class II epitope sequences are alternately underscored. **C-terminal V5 tag**.

Two additional antigen designs were informed by literature reports describing the activity of protein-concatemer fusion vaccines (44, 45): (a) fusion of the 18-epitope concatemer to full-length Com1, a known target of anti-*Coxiella* antibodies (46), to generate a combination vaccine targeting both T cells and B cells; and (b) fusion of the 18-epitope concatemer to *Mycobacterium tuberculosis* HSP70 (MtbHSP70), which has multiple immune-adjuvanting activities that might promote responses to the concatemer epitopes (47, 48). Fusions of the 18-epitope concatemer to the N-terminus or C-terminus of Com1 and to the C-terminus of MtbHSP70, in conjunction with inclusion of the N-terminal TPA signal sequence and the C-terminal V5 expression tag, were evaluated for junctional neoepitopes. Based on the results, construct designs fusing the 18-epitope concatemer to the N-terminus of Com1 and to the C-terminus of MtbHSP70 were selected for production.

#### 3.1.3 Vaccine Constructs

The four vaccine antigen designs were each produced in two different adenoviral vectors (**Table 1**). Constructs in a human Ad5-derived (HuAd5) vaccine vector were produced for murine studies, although these will not be candidates for clinical use due to pre-existing adenovirus immunity in humans. To circumvent such pre-existing immunity, ChAdOx2, a derivative of the chimpanzee adenovirus (ChAd) 68, was utilized as a clinically-relevant vaccine delivery vector (34, 39). In addition, vaccine insert variants that incorporated a Kozak consensus translation initiation sequence were generated in both adenoviral vectors for the QVx18 and QVx27 antigen designs (**Table 1**). Vaccination regimens combining an adenoviral prime vaccine with a heterologous boost using a Modified Vaccinia Ankara (MVA)-vectored vaccine can increase the breadth and robustness of immune responses to a vaccine antigen (42, 49, 50). Therefore, an MVA-vectored vaccine expressing the TPA/27-epitope/V5 concatemer (MVA-kQVx27), which could be used as a heterologous boost for any of the adenovirus vaccines, was also produced.

Western blot detection of the C-terminal V5 expression tag confirmed expression of proteins of the expected sizes from plasmid clones bearing each of the vaccine inserts (**Figures 2B, C** **)**. Vaccine insert expression from the adenoviral constructs was evaluated by flow cytometry, confirming antigen expression from the ChAdOx2 constructs (**Figure 2D**). Notably, the ChAdOx2 variants incorporating the Kozak sequence (kQVx18 and kQVx27) exhibited lower levels of detected antigen expression (**Figure 2D**), as did the HuAd5 constructs (**Figure S3**).

### 3.2 Immunogenicity of Epitope Concatemer Vaccines in Mice

**Figure 3:**
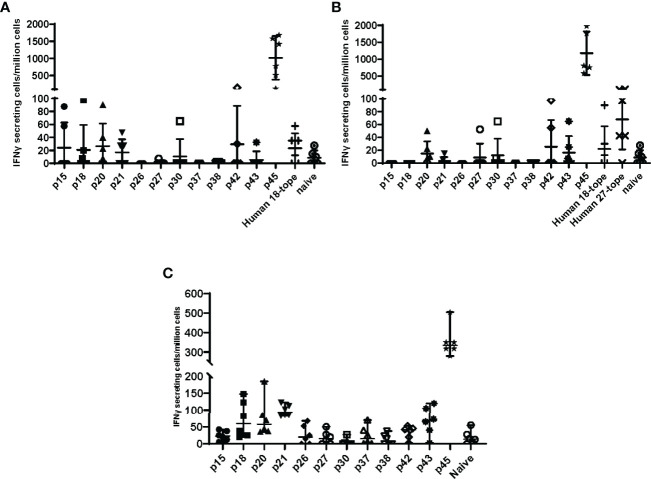
Epitope concatemer vaccine immunogenicity in C57BL/6 mice. Mice received a single dose of **(A)** HuAd5-QVx18 (n=5/group), **(B)** HuAd5-QVx27 (n=5/group), or **(C)** MVA-kQvX27 (n=6/group), administered IM in saline; naïve mice were unvaccinated. Splenocytes were harvested 21 days post-HuAD5 vaccination or 7 days post-MVA vaccination, and tested for peptide-specific T cell responses based on IFNγ production as measured by direct ELISpot assays. Peptides used for stimulation of splenocytes of infected mice are indicated along the X axis. Negative control splenocytes from naïve mice were stimulated with a pool of all assayed peptides. Responses to individual peptides were determined for those epitopes for which antigenic recall responses were observed in *Coxiella*-infected mice (see **Table 4**). Responses to the remaining epitopes were assessed by stimulation with pools of peptides representing epitopes common to both concatemer designs (Human 18-tope) or those unique to the 27-epitope design (Human 27-tope). Data are presented as IFNγ-secreting cells (spot-forming units)/million splenocytes for each individual mouse per group; bars indicate group mean and standard deviation.

To determine if adenoviral delivery of the epitope concatemer vaccines induced T cell responses, the vaccine constructs were tested for immunogenicity in C57BL/6 mice. Mice were vaccinated with HuAd5 vaccines expressing either the QVx18 or QVx27 concatemer (**Table 1**). Splenocytes were harvested at 21 days post-vaccination and tested in ELIspot assays for IFNγ recall responses to twelve peptides for which antigenic recall responses had previously been observed in *Coxiella*-infected C57BL/6 (**Table 4**) (23). Specific responses were detected to 8 of these peptides in at least a subset of vaccinated animals (**Figures 3A, B** **)**. Epitope-specific responses were also detected in C57BL/6 mice immunized with the MVA-kQVx27 vaccine (**Figure 3C**). Responses were strongest to epitope p45. Of note, p45 largely overlaps with one of the class II epitopes included in a peptide vaccine previously reported to confer partial protection against *C. burnetii* infection in C57BL/6 mice (21).

Heterologous prime-boost and prime-boost-boost vaccination schedules were compared using the HuAd5-QVx27 and MVA-kQVx27 vaccine constructs to determine if responses to epitopes other than p45 were increased following boost vaccinations. MVA boost vaccinations were delivered 9 and 12 weeks after the HuAd5 prime vaccination, and animals were sacrificed 7 days after the final MVA boost. The response to p45 remained the dominant response under all vaccination schedules (**Figure S4**). The remaining four adenoviral vaccine constructs (fusion proteins, Kozak sequence addition) were tested to determine whether these specific design variations might support an immunogenic response to a broader range of epitopes. However, results for these four vaccine constructs were similar, with the epitope p45 again eliciting the strongest responses (**Figure S5**).

Based on the combined results of the expression and immunogenicity studies, the QVx18 and QVx27 adenoviral vaccines were selected for additional characterization and testing. The QVx18-Com1 adenoviral vaccines were also evaluated in further studies, to assess the impact of including a known target of anti-*Coxiella* antibodies.

### 3.3 Evaluation of Vaccine Efficacy in a Murine Vaccine-Challenge Model

**Figure 4:**
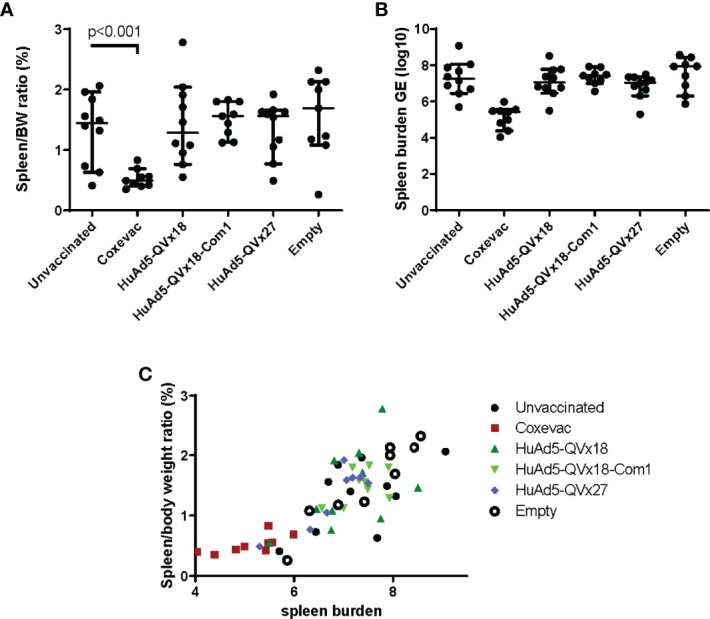
Murine vaccine efficacy study. C57BL/6 mice were vaccinated using a heterologous prime-boost-boost schedule as indicated in **Table 2**. Group labels in the graphs indicate the prime vaccine administered. Sixteen days after the second MVA-kQVx27 boost, mice were intranasally challenged with *C. burnetii* (Nine Mile strain). Mice were euthanized at two weeks after challenge. **(A)** Spleens were harvested and weighed, and spleen-to-body weight ratio determined. Only the Coxevac^®^-immunized group was significantly different from the unvaccinated control group (p<0.001; two-tailed t test). **(B)** Splenocytes were collected and the spleen bacterial burden was determined from homogenates by qPCR. No treatment groups were significantly different from the unvaccinated controls. **(C)** The relationship between spleen-to-body weight ratio and bacterial burden is plotted for each individual mouse.

The QVx18, QVx18-Com1, and QVx27 vaccines were tested in a C57BL/6 mouse vaccine-challenge study, using a HuAd5 prime-MVA boost-boost vaccination schedule (**Table 2**). Satellite immunogenicity groups (3 mice/group; **Table S1**) received a prime HuAd5 vaccination and either two MVA boosts (2 animals) or no boost (1 animal). Epitope-specific responses were confirmed by ELISpot assays, with the strongest responses again being observed against p45 (**Figure S6**). The challenge study was powered to detect an effect size relative to unvaccinated animals approximately 50% of the protective effect of the veterinary vaccine Coxevac^®^ (27). Compared to control animals, Coxevac^®^-immunized animals showed significantly (p<0.001) reduced spleen-to-body weight ratios and bacterial burden upon challenge infection. In contrast, no definitive differences in either of these two disease endpoints were observed between epitope vaccine groups and the unvaccinated control animals (**Figure 4**). These results indicate that a robust T cell response to the p45 epitope alone is not sufficient to confer protection against a subsequent *C. burnetii* infection challenge in this murine infection model. This is consistent with results from other murine vaccine-challenge studies in which multi-epitope T cell responses but not responses to single epitopes conferred protection against *C. burnetii* challenge (21).

### 3.4 Comparison of Vaccine Candidates to a Whole-Cell Control Vaccine in a Guinea Pig Model of Q Fever Vaccine Reactogenicity

The selected ChAdOx2 vaccine candidates and MVA-kQVx27 were evaluated for reactogenicity relative to positive and negative controls (**Table 3**) in a *C. burnetii*-sensitized guinea pig model (41). Induction of anti-*C. burnetii* immune responses were confirmed in sensitized animals prior to vaccine antigen challenge by detection of circulating *C. burnetii*-specific antibodies (**Figure S7**). Sensitization of animals to *Coxiella* antigens was confirmed using the formalin-inactivated phase I whole cell vaccine Coxevac^®^ as a standardized positive control challenge antigen. The Coxevac^®^ challenge sites in *C. burnetii*-sensitized animals developed detectable erythema and induration, with reactions reaching full extent within 24-72 hours post-challenge and persisting through termination on day 7 post-challenge (**Table S2**), consistent with previous observations in this model (41). No gross reactions were noted at Coxevac^®^ challenge sites in saline control animals. No reactions were observed to challenge with a control saline solution in either saline- or *C. burnetii*-sensitized animals.

Reactions to ChAdOx2-vectored vaccines were noted within 8 hours post-challenge in both saline- and *C. burnetii*-sensitized animals; these reactions subsided by day 7 post-challenge (**Table S2**). Minimal reactions were noted to the corresponding empty ChAdOx2 vector. Reactions to the MVA-vectored vaccine and to the empty MVA vector were observed in both sensitized and unsensitized animals, and these reactions persisted through day 7 post-challenge (**Table S2**).

**Figure 5:**
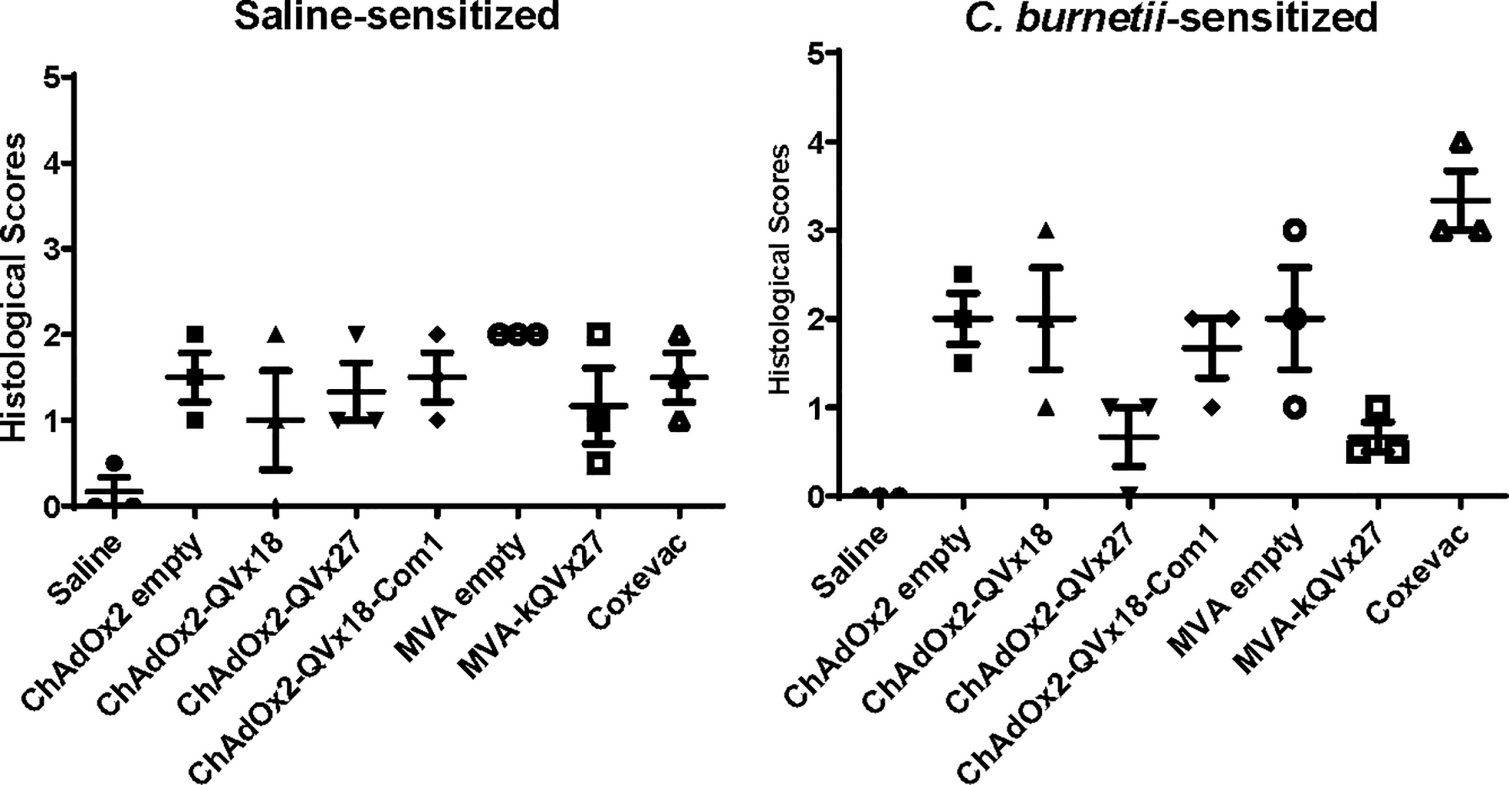
Histological assessment of reactions to vaccine challenge in guinea pigs. Female guinea pigs (n=3/group) were sensitized by intranasal inoculation with 10^6^ GE of *C. burnetii* Nine Mile strain or saline, as described previously (41). Intradermal vaccine challenges (summarized in **Table 3**) are indicated on the X axes. Histological lesions of skin biopsies were scored on a graded scale (41). Data are presented as the mean score from two skin sections per inoculation site for each animal; bars indicate group mean and standard deviation.

Tissues were collected for histological assessment at termination on day 7 post-challenge. No histological reactions were observed at negative control (saline) inoculation sites in either sensitized or unsensitized animals. Significant histological reactions consistent with pyogranulomatous changes were noted at positive control (Coxevac^®^) inoculation sites in sensitized animals (**Figure 5**). Mild to moderate histological changes were observed at inoculation sites for ChAdOx2 or MVA vectored-vaccine candidates in both sensitized and unsensitized animals (**Figure 5**). Similar reactions were observed to empty ChAdOx2 and MVA vectors.

Altogether, these results indicate that reactions occurred to the viral vaccine vectors independent of prior *C. burnetii*-sensitization, and that there was no reactogenicity specific to the presence of the epitope concatemer vaccine antigens in this guinea pig model.

### 3.5 Immunogenicity of Epitope Concatemer Vaccines in Nonhuman Primates

**Figure 6:**
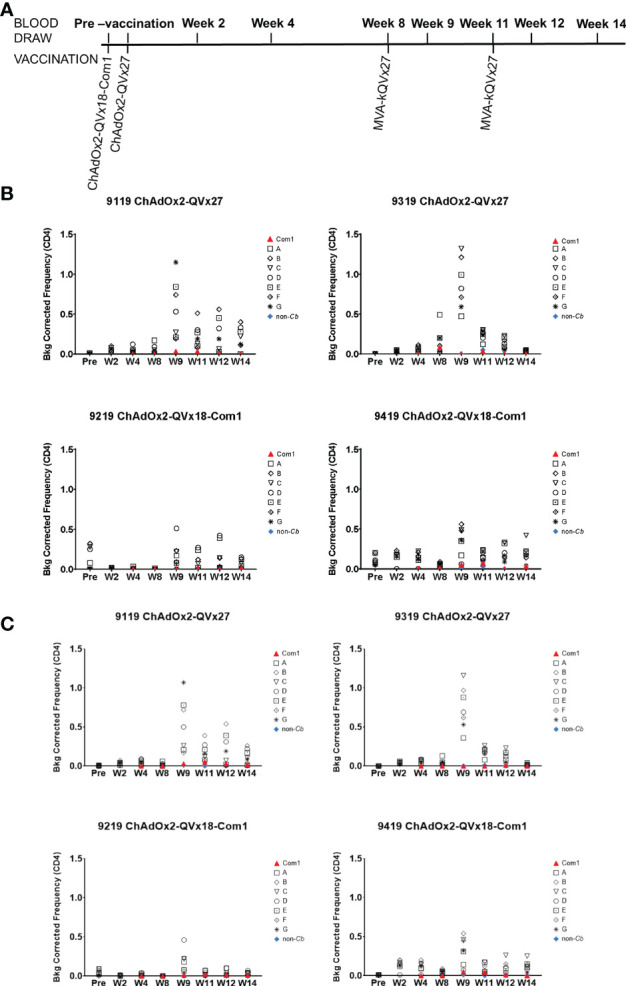
*Ex vivo* CD4^+^ T cell recall responses to vaccine epitopes in vaccinated cynomolgus macaques. **(A)** Schedule of activities for nonhuman primate vaccine immunogenicity study. Cynomolgus macaques received prime vaccinations in Week 0 using the indicated ChAdOx2 vaccine constructs (1x10^9^ IU/vaccination, delivered IM) (n = 2 for each vaccine construct). All animals received MVA-kQVx27 boost vaccinations (1x10^8^ IU/vaccination, delivered IM) at Weeks 8 and 11. Animals 9119 and 9319 received ChAdOx2-QVx27 prime vaccination. Animals 9219 and 9419 received ChAdOx2-QVx18-Com1 prime vaccination. **(B, C)** PBMCs from individual animals were stimulated *ex vivo* by the indicated epitope peptide pools (A-G, see Methods), a pool of peptides representing the full Com1 protein sequence, a short concatemer of non-*Coxiella* peptide sequences (non-*Cb*), or no peptide. T cell activation status following peptide stimulation was evaluated by flow cytometry. Study time points are indicated on the X axis (Pre, pre-vaccination; W, Week). The frequencies of **(B)** CD4^+^ IFNγ^+^ or **(C)** CD4^+^ CD154^+^ IFNγ^+^ T cells are shown as a percentage of total CD4^+^ T cells (Y axis). Data shown are background corrected (peptide stimulated minus unstimulated). Individual macaque identification numbers and prime vaccine are noted in each graph title.

Based on the results of the mouse immunogenicity studies of the epitope concatemer vaccines, two vaccine candidates (ChAdOx2-QVx18-Com1 and ChAdOx2-QVx27) were advanced to initial immunogenicity studies in Mauritian cynomolgus macaques (n=2 per group), using a ChAdOx2 prime-MVA boost-boost vaccination schedule (**Figure 6A**) to maximize the possibility of observing immunogenic responses. Three of the animals shared a single major histocompatibility complex (MHC) haplotype, and one animal (9219) was homozygous for this haplotype (**Table S3**). The MVA-kQVx27 vaccine, which included all the epitopes in the concatemers expressed by both prime vaccines (see **Tables 1**, **5**), was used for all heterologous boost vaccinations. Animals were visually examined twice per week for any signs of injection site reactions. No reactions were observed following the prime vaccinations. After the first MVA boost all animals exhibited a minor transient weight loss (**Figure S8**).

**Figure 7:**
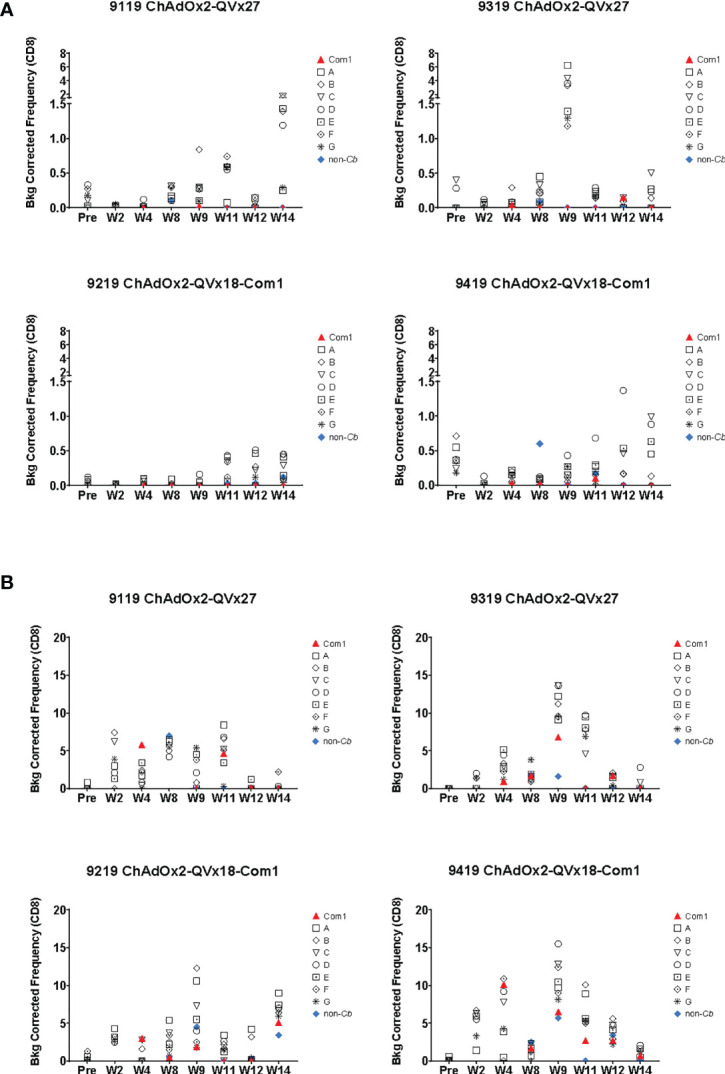
*Ex vivo* CD8^+^ T cell recall responses to vaccine epitopes in vaccinated cynomolgus macaques. Study time points are indicated on the X axis (Pre = pre-vaccination; W = Week); see **Figure 6A** for the corresponding vaccination schedule. Individual macaque identification numbers and prime vaccine are noted in each graph title. MVA-kQVx27 boost vaccinations were administered at Week 8 and Week 11. PBMCs from individual animals were stimulated *ex vivo* by indicated epitope peptide pools (A-G, see Methods), a pool of peptides representing the full Com1 protein sequence, a short concatemer of non-*Coxiella* peptide sequences (non-*Cb*), or no peptide. T cell activation status following peptide stimulation was evaluated by flow cytometry. The frequencies of **(A)** CD8^+^ IFNγ^+^ or **(B)** CD8^+^ T-bet^+^ T cells are shown as a percentage of total CD8^+^ T cells (Y axis). Data shown are background corrected (peptide stimulated-unstimulated).

Antigen-specific CD4^+^ T cell responses were observed in all animals one week after the first boost, as indicated by the increased frequency of CD4^+^ T cells expressing IFNγ and the early activation marker CD154 in response to peptide stimulation, (see week 9 in **Figures 6B, C** **)**. CD4^+^ T cells expressing the transcription factor T-bet were also increased following immunization (**Figure S9**), paralleling the contribution of T-bet to responses to inactivated whole cell *C. burnetii* vaccines (11, 18). Antigen-specific CD8^+^ T cell responses were also observed, reflected by increased frequencies of both CD8^+^ IFNγ^+^ (**Figure 7A**) and CD8^+^ T-bet^+^ (**Figure 7B**). The two animals receiving the ChAdOx2-QVx27 prime vaccination showed greater responses than did the two primed with the ChAdOx2-QVx18-Com1 vaccine, though determining whether this reflects true differences in prime vaccine function or simple variations in responses by individual macaques would require a larger study.

Responses were observed to multiple peptide pools in all animals, indicating that cynomolgus macaques exhibit responses to a broader range of the vaccine-encoded human-targeted HLA class II epitopes than were observed in C57BL/6 mice. These results demonstrate that delivery of human T cell epitope concatemers *via* viral vaccine vectors can elicit T cell responses to multiple epitopes in non-human primates, although the most effective vaccination schedule, the duration of the induced T cell responses, and the magnitude of any recall response upon infection challenge remain to be determined.

## 4 Discussion

We describe here the design and initial preclinical testing of multi-epitope T cell-targeted vaccine candidates for the prevention of Q fever disease in humans. Promiscuous HLA class II epitope clusters included in these vaccines were selected from *C. burnetii* antigens to which the host immune system is expected to be exposed during course of infection (23). Although the specific roles in the pathophysiology of infection are not known for many of these antigens, the durable T cell memory responses detected 7-10 years after infection associate the selected epitopes with presumed post-infection immunity. Whether exposure to these epitopes separately from an active infection can confer protection against subsequent Q fever disease remains to be determined, and a key objective of the studies reported here was assessment of potential correlates of immune response, safety and efficacy in established experimental animal models of *C. burnetii* infection (51).

C57BL/6 mice were selected for use in initial vaccine immunogenicity and efficacy tests, as they exhibit less severe disease and lower mortality in response to *C. burnetii* infection compared to other mouse strains (52), and are arguably more reflective of the acute non-lethal disease observed in humans. Twelve of the twenty-seven human epitopes included in these vaccines stimulated antigenic recall responses in splenocytes from *C. burnetii*-infected C57BL/6 mice (**Table 4** and **Figure 1**). However, vaccine immunogenicity in the C57BL/6 mouse model was dominated by a single epitope (p45), and this was insufficient to confer protection against infection challenge. Whether the narrow immunogenicity of these vaccines in C57BL/6 is due to suboptimal recognition of the human-targeted epitopes in the C57BL/6 MHC background, to poor processing and presentation of the epitope concatemers, or to other factors remains unknown. In this context, three observations suggest that the limited immunogenicity is not due simply to truncated translation of the vaccine antigens: Expression of full-length concatemers in cultured cells was confirmed by immunological detection of the C-terminal V5 expression tag (**Figure 2**). The dominant p45 epitope is in the center of the epitope concatemers (see **Table 5** and **Figures S1, S2**), and there is no apparent bias toward higher immunogenicity of N-terminal epitopes. In addition, mice vaccinated with the HuAd5-QVx18-Com1 vaccine, in which Com1 is C-terminal to the epitope concatemer, exhibit robust T cell responses to Com1 (**Figure S5**).

Guinea pigs played a central role in the identification of the causative infectious agent for Q fever (53), and they more fully replicate aspects of human acute Q fever than do mice (51, 54). Unfortunately, reagents and protocols for analyzing cellular immunology in this species are limited, and little information is available regarding recognition of human T cell epitopes by the guinea pig immune system. However, the guinea pig is an established model for assessing the reactogenicity of candidate Q fever vaccines (41, 55). We therefore tested the T cell vaccine candidates in a sensitized guinea pig reactogenicity model. The observation of transient reactions to the viral vectors (ChAdOx2 and MVA) in both sensitized and unsensitized animals is consistent with the transient reactions reported in human tests of ChAd- and MVA-vectored vaccines expressing non-*Coxiella* antigens (56, 57). While the results indicate that the antigens encoded in the vaccine candidates do not elicit specific hypersensitivity responses, we note that immunogenicity of these antigens in guinea pigs was not directly demonstrated. As the recognition of the encoded epitopes by guinea pig MHCs is unknown, we cannot definitively rule out the potential for specific reactogenic responses in a different species.

Aerosol exposure models of acute Q fever have been established in both rhesus and cynomolgus macaques, and the latter have been suggested as a preferred non-human primate model on the basis of higher similarity of pathological responses to those in humans (58, 59). Macaque MHC classes are homologous to those of humans, although they do not exhibit simple one-to-one correlation with human HLA subtypes due to the more extensive expansion of MHC genes in macaques (60). Notably, the repertoire of MHC class II epitopes from *Mycobacterium tuberculosis* recognized in both cynomolgus and rhesus macaques largely overlaps that of human T cells (61). Thus, macaque cellular responses to T cell vaccines are expected to be more predictive of human responses than are those of the small animal models. We advanced two of our vaccine candidates to an initial immunogenicity study in cynomolgus macaques. Specific CD4^+^ responses following vaccination were observed to multiple peptide pools in all animals, which had distinct MHC genotypes, indicating that cynomolgus macaques exhibit responses to a broader range of the human-targeted HLA class II epitopes encoded in these vaccines than was observed in C57BL/6 mice. Intriguingly, specific CD8^+^ responses were also observed in vaccinated macaques. Whether these responses arise from cryptic MHC class I epitopes within the vaccine-encoded class II sequences or from unconventional MHC class II restricted CD8^+^ T cells (62) remains to be investigated.

Defining an appropriate development path for vaccines expected to demonstrate efficacy only in the context of the human immune system has been recognized as a limitation in emerging disease response readiness, requiring consideration of regulatory requirements on a case-by-case basis (63, 64). In particular, species differences in MHC binding preferences present challenges in the preclinical development of human-targeted T cell vaccines, especially for a select agent such as *C. burnetii* for which intentional human exposure would not be considered safe or ethical (65, 66), as vaccine responses in animal models may not fully reflect the potential responses to such vaccines in humans. These challenges are reflected in the studies reported here for human-targeted multi-epitope Q fever vaccines. The lack of established tools for cellular immunology limits the utility of the guinea pig in defining correlates of immunity or protection for development of such T cell-targeted vaccines. The greater availability of immunological reagents and relatively low cost have made the mouse a favored research model for Q fever as for other diseases. However, the combined results of our murine immunogenicity and vaccine-challenge studies indicate that the C57BL/6 mouse is not an effective model for preclinical testing of these specific human-targeted multi-epitope vaccines.

The broad immunogenic responses to the vaccine candidates in cynomolgus macaques provide a foundation for further preclinical and clinical evaluation of a T cell-targeted vaccine for Q fever. Reagents and protocols are established for assessment of both humoral and cellular immune responses in macaques, enabling animal efficacy studies capable of defining correlates of protection bridging animal model results with human clinical responses. In this context, some consideration of the design of animal efficacy studies is warranted. Sterilizing immunity is a common efficacy objective in vaccine studies and could be reasonably considered a correlate of protection from disease. However, in the aerosol-challenge macaque model, vaccination with the efficacious commercial vaccine Q-VAX reduced but did not prevent bacteremia, though disease symptoms and pathology were reduced or abrogated in vaccinated animals compared to unvaccinated animals (59). Thus, prevention of acute Q fever disease may not require fully sterilizing immunity, and requiring an endpoint in the macaque model that is not reached by a vaccine with proven efficacy in humans may represent an unachievably stringent objective for development of a new Q fever vaccine. The suitability of sterilizing immunity as an efficacy endpoint is particularly questionable for T cell-targeted vaccines intended to induce infection-clearing, and thereby disease-limiting, cellular immunity rather than infection-blocking humoral immunity. For novel vaccine development programs anticipating such challenges, the US Food and Drug Administration recommends early discussions with agency staff regarding the design of studies that would support regulatory review (66). The immunogenic responses in macaques to the vaccine candidates described here provide a basis for such a discussion to define an expanded preclinical testing plan that could inform and justify first-in-human testing of a T cell-targeted vaccine for Q fever.

## Data Availability Statement

The original contributions presented in the study are included in the article/**Supplementary Material**. Further inquiries can be directed to the corresponding authors.

## Ethics Statement

The animal study was reviewed and approved by Colorado State University Institutional Animal Care and Use Committee; Massachusetts General Hospital Institutional Animal Care and Use Committee; Oxford University Animal Care and Ethical Review Committee; US Defense Threat Reduction Agency Animal Care and Use Review Office.

## Author Contributions

AES, SRP, LM, LB, AS, PR, RB, AP, AG, CR, and MP conceptualized and designed the study and experiments. Experiments were performed by SRP, CD, GR, LS-R, and LB. Data were analyzed and interpreted by AES, SRP, LM, CD, GR, LB, AS, PR, CR, and MCP. GR, LM, PR, RB, ADG, CR, and MP contributed critical reagents, materials and analytic tools. AES, LM, AG, RB, ADG, CR, and MP acquired funding and supervised research activities. AES, SRP, LM, LB and CR wrote the manuscript and GR, PR, AS, AP, AG, RB, and MP critically reviewed and approved the manuscript. All authors contributed to the article and approved the submitted version.

## Funding

This research was supported by contract HDTRA1-15-C-0020 from the US Defense Threat Reduction Agency (www.dtra.mil), awarded to Massachusetts General Hospital (MCP Lead Principal Investigator); work by authors at other institutions was supported by subcontracts under the prime contract award to MGH.

## Conflict of Interest

MP, AES, ADG, LM, AG, and AS are named inventors on patent application WO 2019/183627 A1, *“Coxiella burnetii* epitopes for T cell-targeted Q fever vaccines”.

AG is a senior officer and shareholder and AS was an employee of Innatoss Laboratories B.V., which provides diagnostic screening for Q fever. ADG is a senior officer and shareholder, and LM and GR are employees of EpiVax, Inc., a company specializing in immunoinformatic analysis. Innatoss Laboratories B.V and EpiVax, Inc., own patents to technologies utilized by associated authors in the research reported here.

AP is Chair of UK Dept. Health and Social Care’s (DHSC) Joint Committee on Vaccination & Immunisation (JCVI), and is a member of the WHO’s SAGE. AP is an NIHR Senior Investigator. The views expressed in this article do not necessarily represent the views of DHSC, JCVI, NIHR or WHO. CR, CD, and AP are named inventors on a patent application in the field of meningococcal vaccines. AP waives all his rights to any patent.

The remaining authors declare that the research was conducted in the absence of any commercial or financial relationships that could be construed as a potential conflict of interest.

## Publisher’s Note

All claims expressed in this article are solely those of the authors and do not necessarily represent those of their affiliated organizations, or those of the publisher, the editors and the reviewers. Any product that may be evaluated in this article, or claim that may be made by its manufacturer, is not guaranteed or endorsed by the publisher.
