# Supplementary material for: Evaluation of a Human T Cell-Targeted Multi-Epitope Vaccine for Q Fever in Animal Models of Coxiella burnetii Immunity
Source: Front Immunol. 2022 May 16;13:901372. doi: 10.3389/fimmu.2022.901372 (PMC9149306; doi:10.3389/fimmu.2022.901372)
Supplement: Supplementary file 1 [file DataSheet_1.pdf]

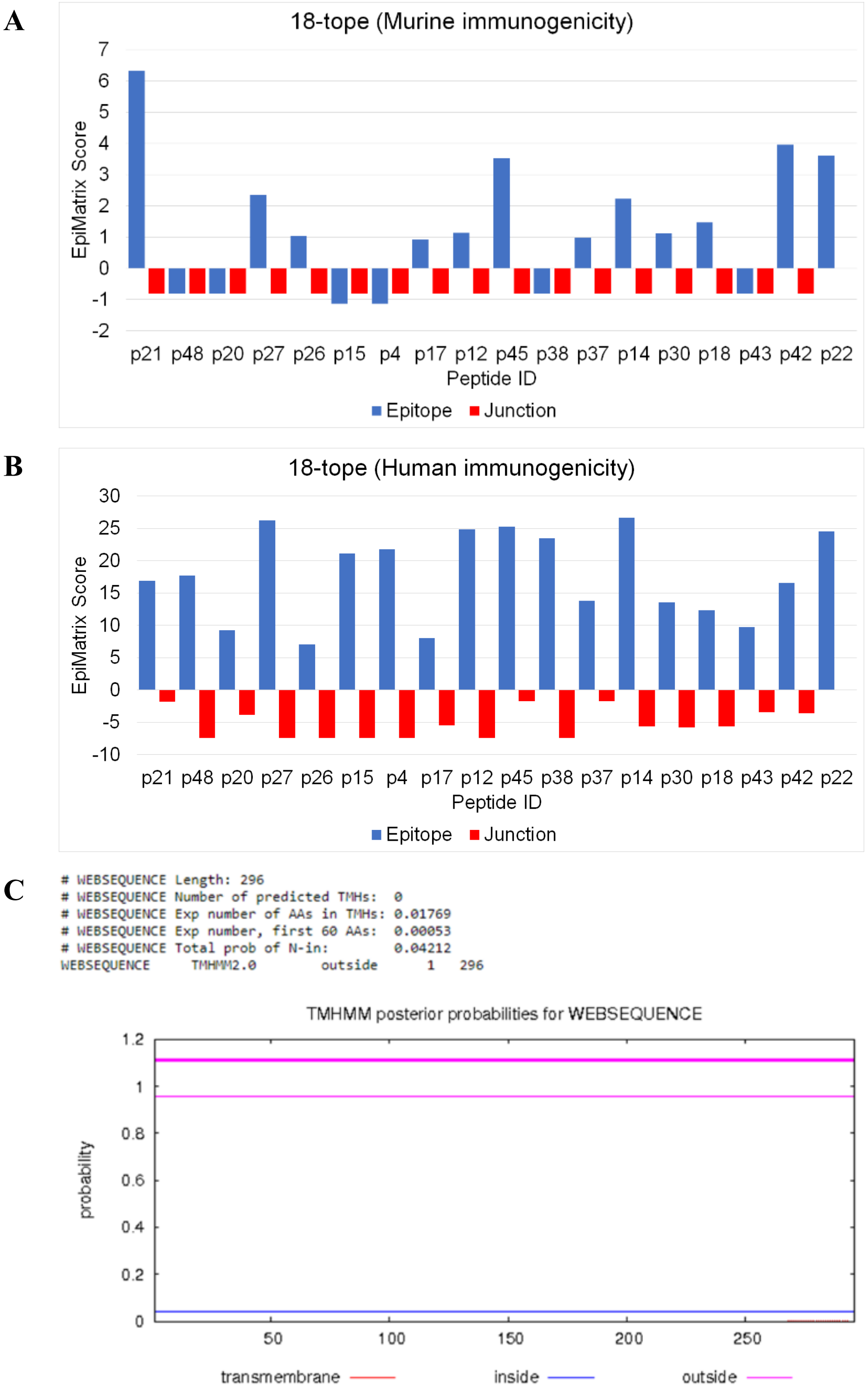

Figure S1. Evaluation of 18 epitope concatemer design. (A) No significant junctional immunogenicity predicted by VaxCAD for murine MHC class II I-Ab-restricted epitopes. VaxCAD analyzes immunogenicity potential across a sliding window of specified amino acid length; higher EPX scores indicate higher immunogenicity potential. (B) No significant junctional immunogenicity predicted by VaxCAD for human HLA class II-restricted epitopes (DRB*0101, *0301, *0401, *0701, *0801, *0901, *1101, *1301, *1501). (C) No transmembrane domain predicted by TMHMM v 2.0.

A

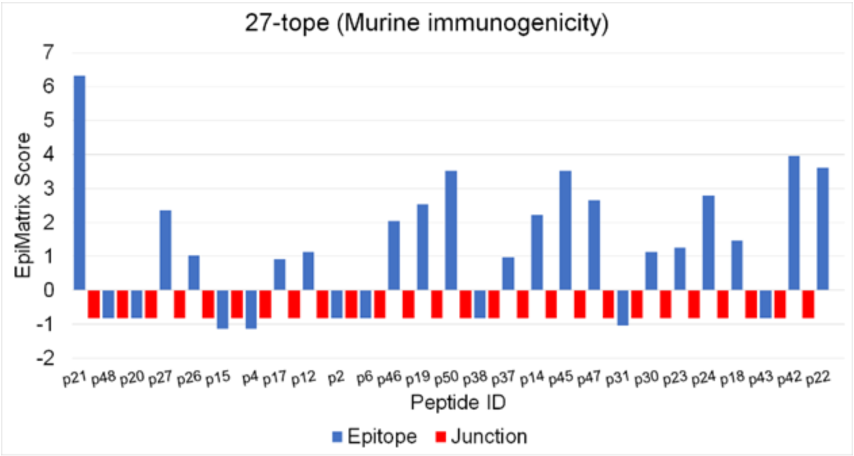

B

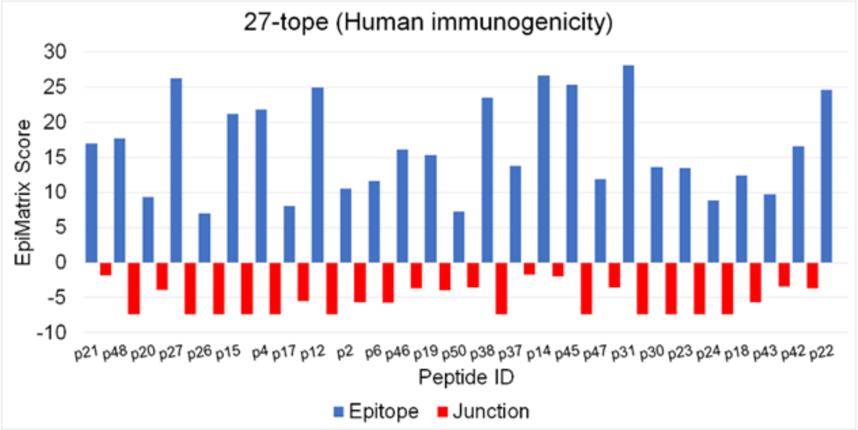

C

```
# WEBSEQUENCE Length: 445
# WEBSEQUENCE Number of predicted TMHs:  0
# WEBSEQUENCE Exp number of AAs in TMHs: 0.0143
# WEBSEQUENCE Exp number, first 60 AAs:  0.00049
# WEBSEQUENCE Total prob of N-in:        0.00688
WEBSEQUENCE     TMHMM2.0        outside      1    445
```

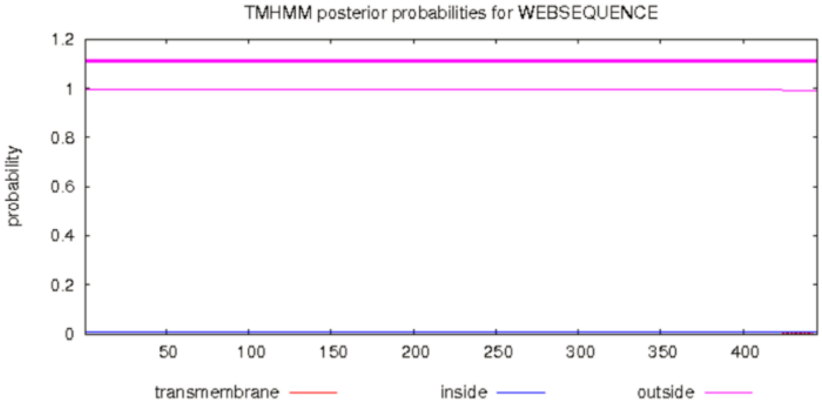

Figure S2. Evaluation of 27 epitope concatemer design. (A) No significant junctional immunogenicity predicted by VaxCad for murine MHC class II I-Ab-restricted epitopes. VaxCAD analyzes immunogenicity potential across a sliding window of specified amino acid length; higher EPX scores indicate higher immunogenicity potential. (B) No significant junctional immunogenicity predicted by VaxCad for human HLA class II-restricted epitopes (DRB*0101, *0301, *0401, *0701, *0801, *0901, *1101, *1301, *1501). (C) No transmembrane domain predicted by TMHMM v 2.0.

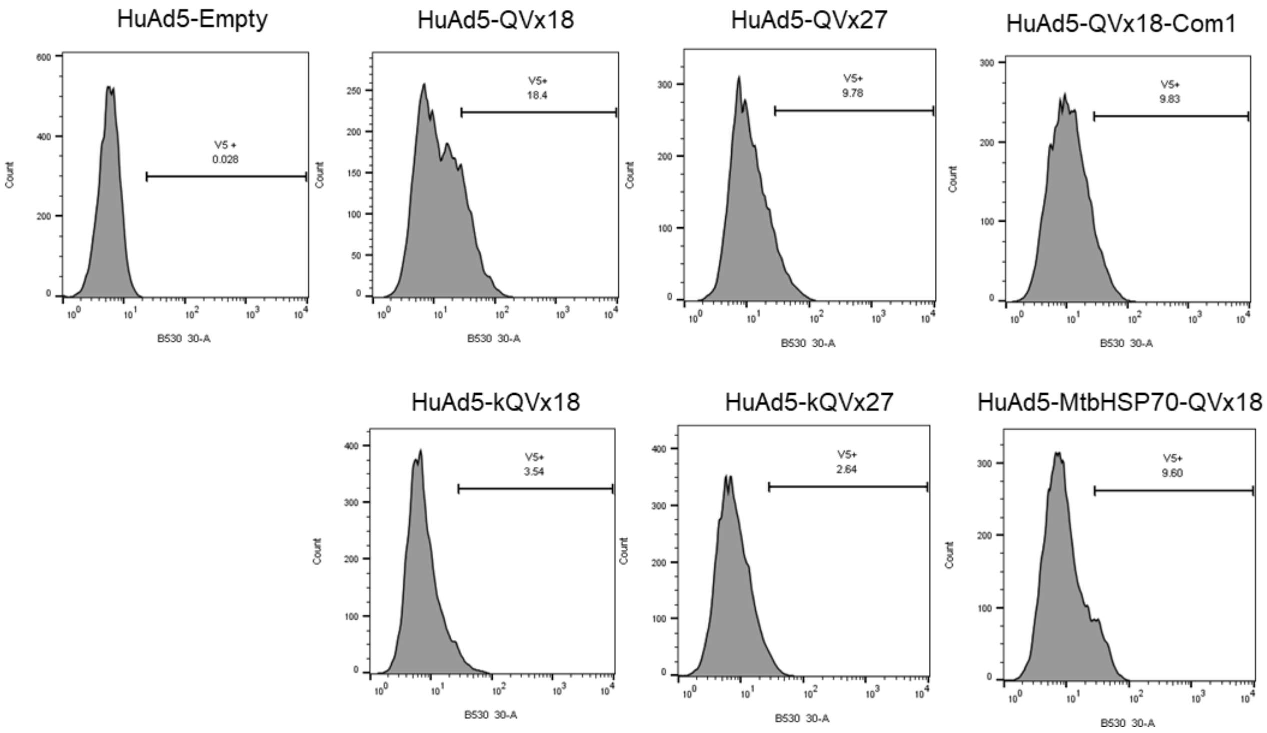

Figure S3. Expression of multi-epitope antigens from HuAd5 vaccine constructs. Expression of vaccine antigens in HeLa cells infected with HuAd5 vaccine constructs detected by anti-V5 flow cytometry.

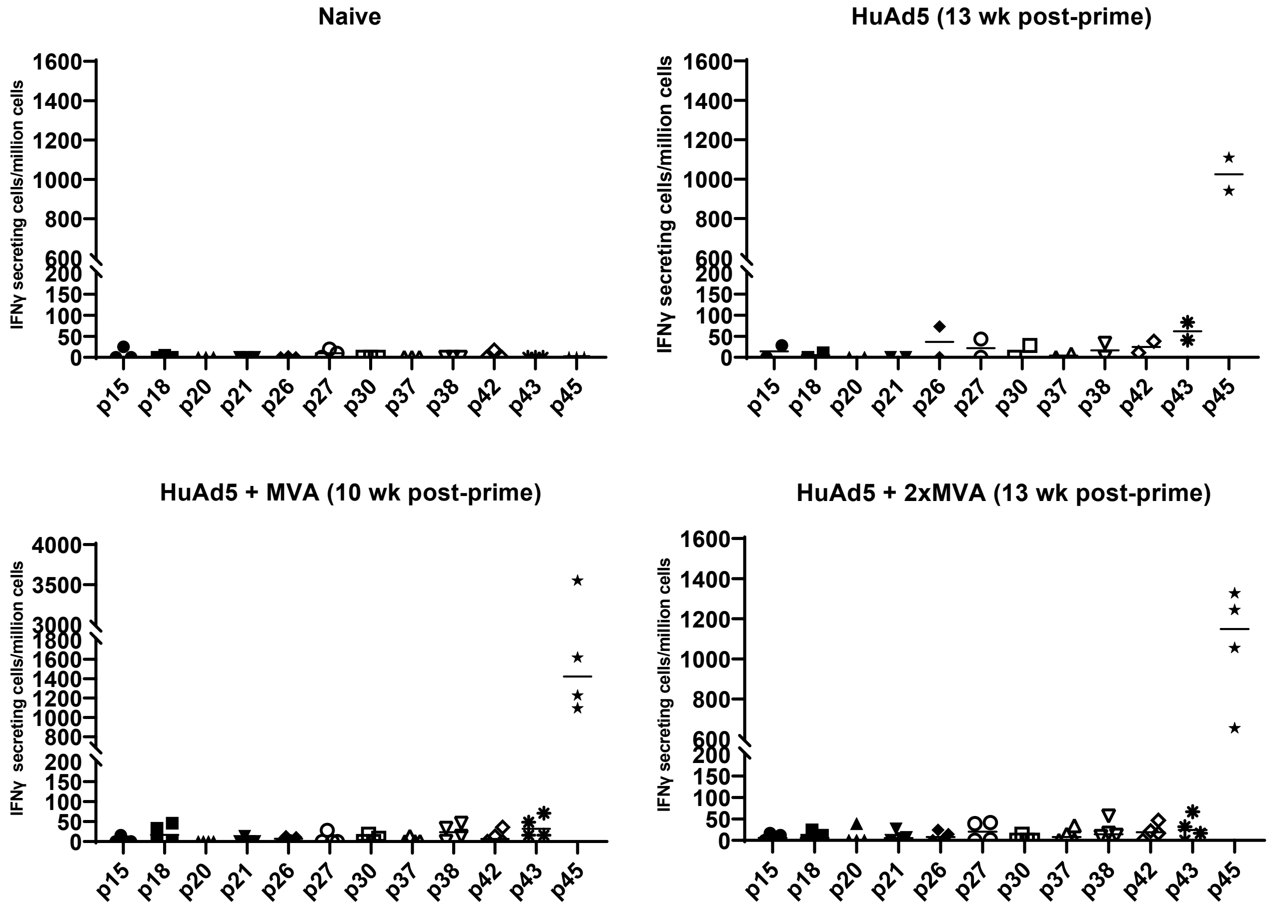

Figure S4. QVx27 concatemer vaccine immunogenicity in C57BL/6 mice. Mice (n=10/group) received the HuAd5-QVx27 vaccine (10^9 IU) IM in saline. MVA-kQVx27 boost vaccinations (10^7 IU) were delivered 9 weeks (8 animals) and 12 weeks (4 animals) after the HuAd5 prime vaccinations. Splenocytes were harvested 7 days after the final MVA boost vaccination from 4 animals following each of the two boost vaccinations. Splenocytes were also harvested from a prime vaccination only group (2 animals, 13 weeks after prime vaccination) and an unvaccinated negative control group (3 animals). Splenocytes were tested for peptide-specific T cell responses based on IFNγ production as measured by direct ELISpot assays following stimulation with the indicated individual peptides. Data points for individual animals are shown with the bar indicating group mean for each stimulation condition.

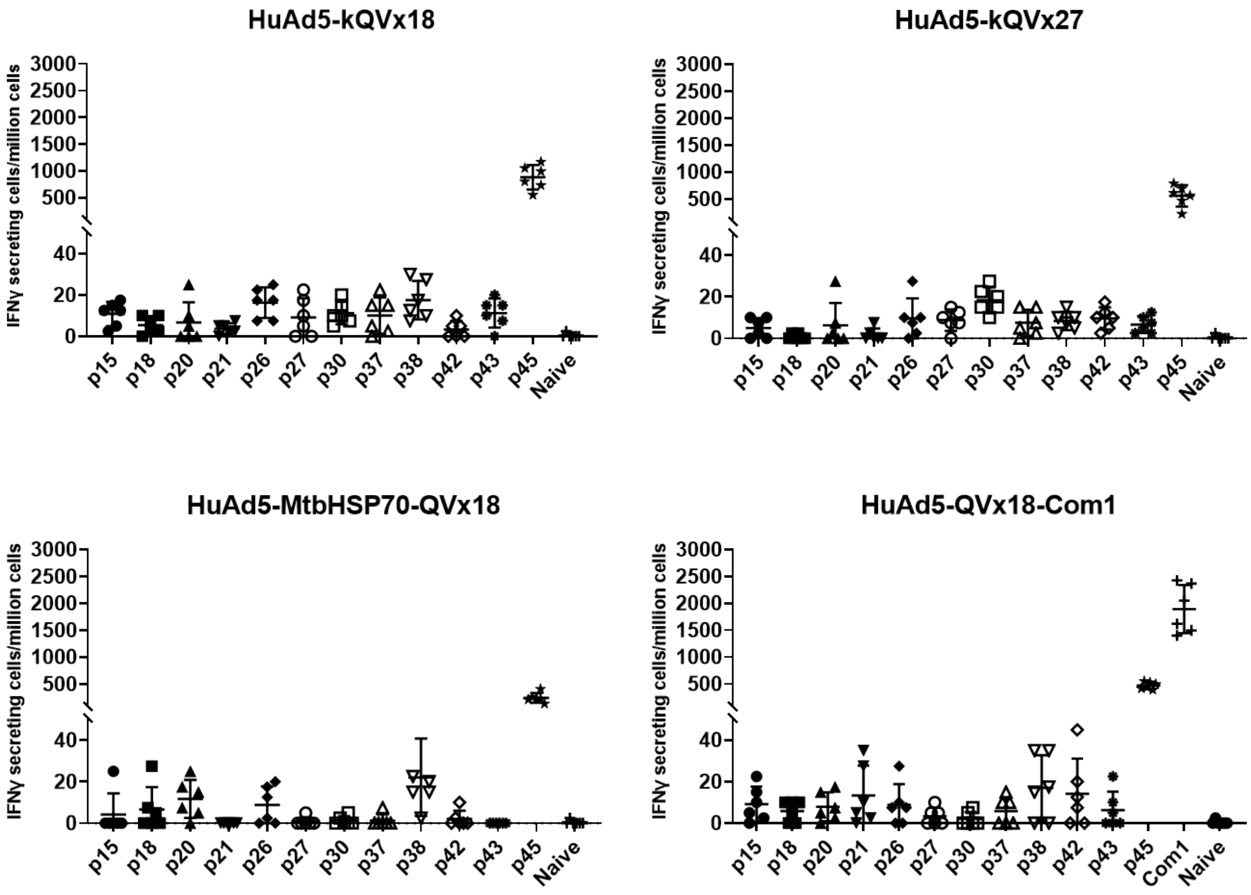

Figure S5. Epitope concatemer vaccine immunogenicity in C57BL/6 mice. Mice (n=5/group), received the indicated HuAd5 vaccines IM in saline (10^9 IU). Splenocytes were harvested 21 days post-vaccination and tested for peptide-specific T cell responses based on IFNγ production as measured by direct ELISpot assays following stimulation with the indicated individual peptides or with a pool of peptides representing the full Com1 protein sequence. Negative control splenocytes from naïve mice were stimulated with a pool of all assayed peptides. Data points for individual animals are shown for each stimulation condition; error bars represent group mean and standard deviation.

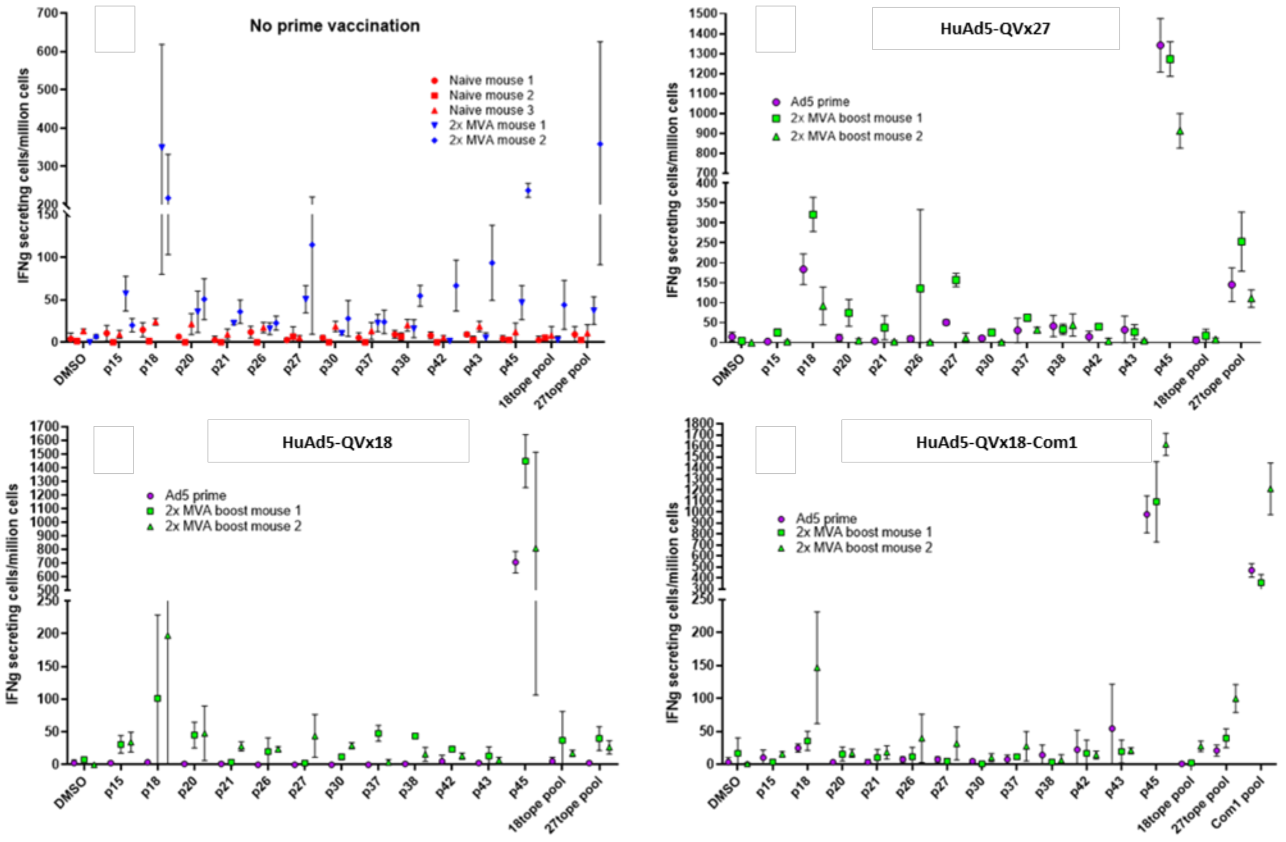

Figure S6. Epitope-specific responses in mice following prime (HuAd5)-boost (MVA) vaccination. C57BL/6 mice received vaccines as indicated in **Table S1**, delivered IM in saline; the prime vaccination administered is indicated at the top of each data graph. Splenocytes were harvested 14 days following the second MVA boost and tested for peptide-specific T cell responses based on IFNγ production measured by direct ELISpot assays. Peptides for which responses had previously been observed in mice were assayed individually (2 µg/mL). The remaining human-targeted peptides for each vaccine design were assayed as peptide pools (2 µg/mL) representing epitopes common to both concatemer designs ("18tope pool") or those unique to the 27-epitope design ("27tope pool"). The "Com1 pool", composed of a series of overlapping peptides covering the full-length Com1 protein sequence, was assayed at a concentration of 6 µg/mL. Splenocytes from all animals were viable and capable of responding to non-specific stimulation by 2 µg/mL Concanavalin A (data not shown). Data for each animal are presented as the mean and standard deviation of triplicate assays.

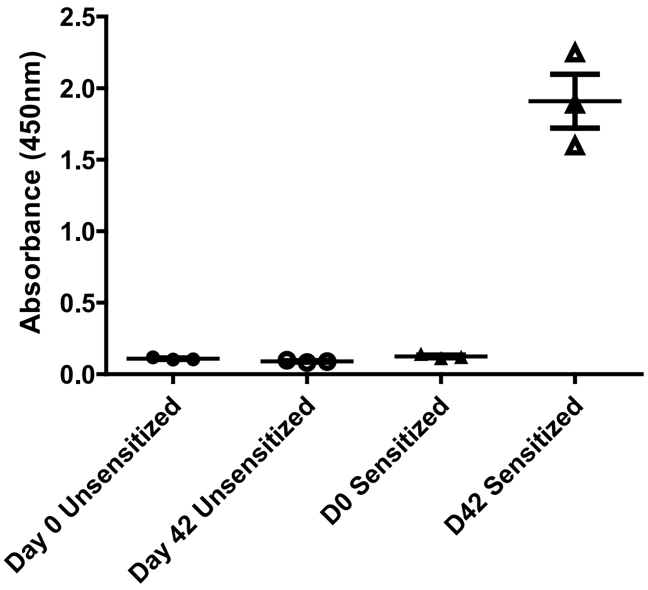

Figure S7. Serological status of guinea pigs in reactogenicity study. Animals were sensitized via infection with the Nine-Mile strain of *C. burnetii* (10^6 GE delivered intranasally) or PBS (n=3 per group). Blood samples were collected on study Day 0 (prior to infection for sensitization) and on Day 42 (prior to antigen challenge). Levels of circulating anti-*Coxiella* antibodies were assessed by ELISA (IDEXX Q Fever antibody test kit). Error bars represent group mean and standard deviation.

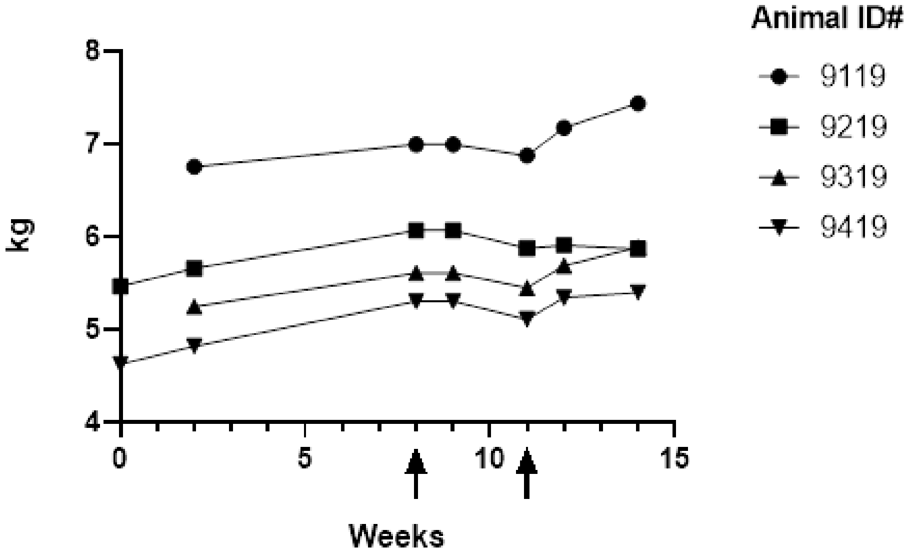

Figure S8. Body weights of cynomolgus macaques during vaccine immunogenicity study. See **Figure 6** for the full vaccination and blood draw schedule. Arrows on X axis indicate time of boost vaccinations. Animals were weighed at the indicated study timepoints. Macaques 9119 and 9319 received ChAdOx2-QVx27 prime vaccination. Macaques 9219 and 9419 received ChAdOx2-QVx18-Com1 prime vaccination. MVA-kQVx27 was administered for all boost vaccinations.

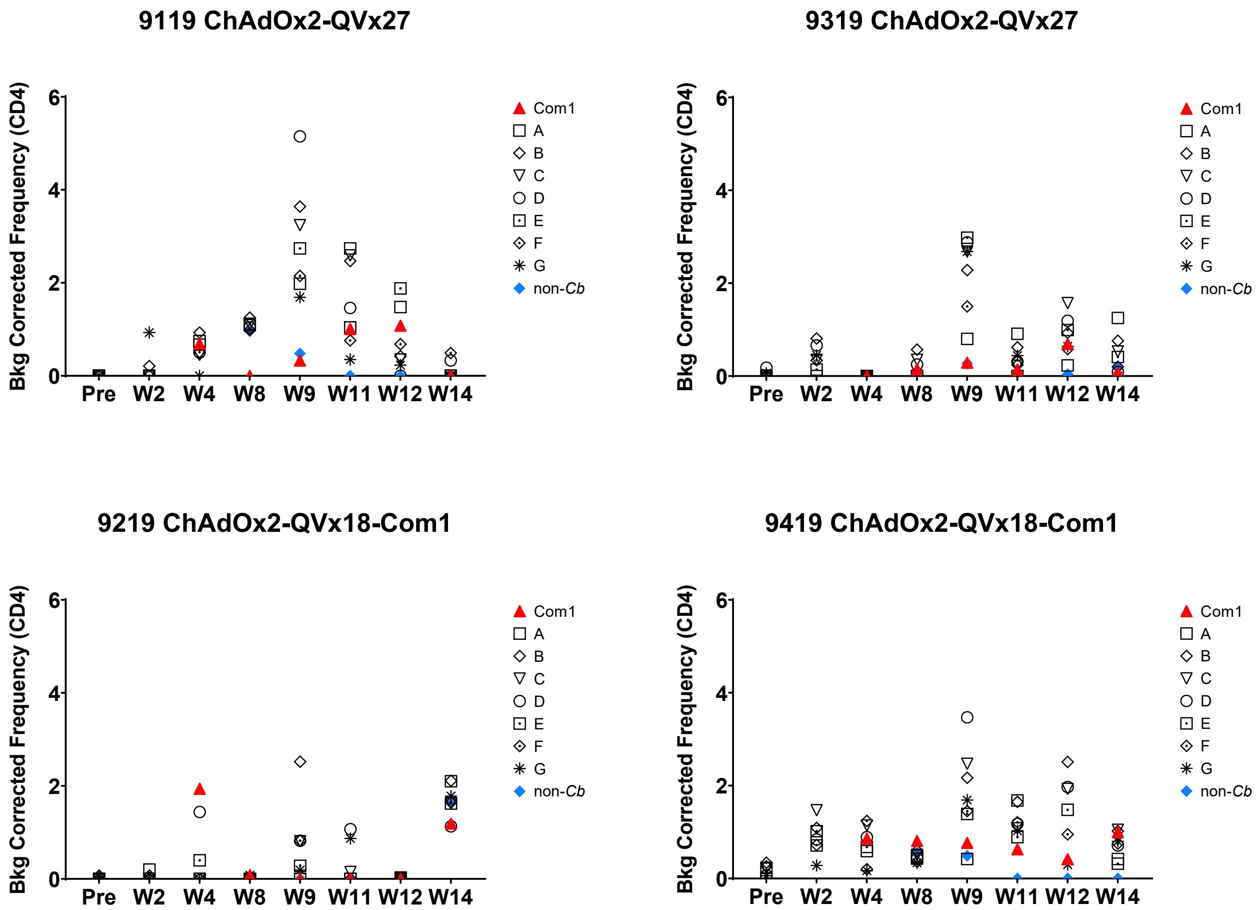

Figure S9. ***Ex vivo*** **CD4$^+$ T-bet$^+$ T cell responses to vaccine epitopes in cynomolgus macaques.** Study time points are indicated on the X axis (Pre = pre-vaccination; W = Week); see **Figure 6** for the corresponding vaccination schedule. Individual macaque identification numbers and prime vaccine are noted in each graph title. MVA-kQVx27 boost vaccinations were administered at Week 8 and Week 11. PBMCs from individual animals were stimulated *ex vivo* by indicated epitope peptide pools (A-G, see Methods), a pool of peptides representing the full Com1 protein sequence, a short concatemer of non-*Coxiella* peptide sequences (non-*Cb*), or no peptide. T cell activation status following peptide stimulation was evaluated by flow cytometry. The frequencies of CD4$^+$ T-bet$^+$ T cells are shown as a percentage of total CD4+ T cells (Y axis). Data shown are background corrected (peptide stimulated-unstimulated).

Table S1. Satellite immunogenicity groups for murine challenge study (C57BL/6). Virally-vectored vaccines were administered IM. Each dose was delivered as 50 µL in each hind leg (100 µL total), for a total of 10^9 IU of each of the HuAd-vectored vaccines. Each MVA-27tope boost was a total of 10^7 IU.

Prime Day 0	Boost Day 56	Boost Day 77	Euthanize Day 91	Mice (N)
PBS (100 µL total)	None	None	ELISpot	3
HuAd5-QVx18	MVA- QVx27	MVA- QVx27	ELISpot	2
	None	None	ELISpot	1
HuAd5-QVx18-Com1	MVA- QVx27	MVA- QVx27	ELISpot	2
	None	None	ELISpot	1
HuAd5-QVx27	MVA- QVx27	MVA- QVx27	ELISpot	2
	None	None	ELISpot	1
None	MVA- QVx27	MVA- QVx27	ELISpot	2

Table S2. Gross reactions to vaccine challenge in guinea pigs. Intradermal challenge inoculation sites were evaluated at 3 and 8 hr post injection and daily thereafter for 7 days. Reactions are recorded for each day post-challenge (DPC), using the following notations: I=induration; E=Erythema, e=slight discoloration; d=diffuse erythema (no discrete circle); P=painful to touch; NR=no reaction; ND=not determined. The diameter of erythema and thickness of induration of each inoculation site were measured with calipers.

A. Unsensitized animals.

**Post-challenge timepoint**	**Challenge Inoculation Site**							
	**Saline**	**Coxevac 1ug**	**ChAdOx2-QVx18**	**Empty ChAdOx2**	**ChAdOx2-QVx27**	**ChAdOx2-QVx18-Com1**	**MVA-kQVx27**	**Empty MVA**
**Animal GP1**								
~3 hr	NR	NR	NR	NR	NR	NR	NR	NR
8 hr	NR	NR	e 5mm	NR	e 5mm	e 5mm	e 5mm	e 5mm
1	NR	NR	e 5mm	NR	e 5mm	e 10mm	e 5mm	eI 8-10mm
2	NR	NR	e 5mm	NR	e 5mm	e 10mm	e 8-10mm	eI 8-10mm
3	NR	NR	ed	NR	ed	e 10mm	EI 8-10mm	EI 10-11mm
4	NR	NR	NR	NR	ed	e 8mm	eI 6-7mm	EI 8-9mm
5	NR	NR	NR	NR	ed	e 8mm	ei 6-7mm	EI 8mm
6	NR	NR	NR	NR	ed	ed	ei 6-7 mm	EI 7mm
7	NR	NR	NR	NR	NR	NR	ei 5mm	ei 6mm
**Animal GP2**								
~3 hr	NR	NR	e 6mm	NR	NR	NR	NR	e 5mm
8 hr	NR	NR	e 6mm	NR	NR	NR	e 5mm	e 5mm
1	NR	NR	e 10mm	NR	NR	e 5mm	e 5mm	eI 6-8mm
2	NR	NR	e 10mm	NR	NR	e 5mm	eI 5mm	eI 8-10mm
3	NR	NR	edI	NR	NR	NR	ei 5mm	EI 8mm

4	NR	NR	edI	NR	NR	NR	e 5mm	ed 8mm
5	NR	NR	edI	NR	NR	NR	ed	ed 8mm
6	NR	NR	edI	NR	NR	NR	ed	ed 7mm
7	NR	NR	edI	NR	NR	NR	ed	ed 5mm
**Animal GP3**								
~3 hr	NR	NR	NR	NR	NR	NR	NR	NR
8 hr	NR	NR	NR	NR	NR	NR	NR	e 5mm
1	NR	NR	e 5mm	NR	ed	ed	NR	e 5mm
2	NR	NR	ei 5mm	NR	e 5mm	ed	NR	eI 10mm
3	NR	NR	ei 5mm	NR	NR	NR	NR	eI 10mm
4	NR	NR	ei 5mm	NR	NR	NR	NR	eI 10mm
5	NR	NR	ei 5mm	NR	NR	NR	NR	ei 10mm
6	NR	NR	ei 5mm	NR	NR	NR	NR	ei 6mm
7	NR	NR	ei 2mm	NR	NR	NR	NR	ei 5mm

B. *C. burnetii*-sensitized animals

Post-challenge timepoint	Challenge Inoculation Site							
	Saline	Coxevac 1ug	ChAdOx2-QVx18	Empty ChAdOx2	ChAdOx2-QVx27	ChAdOx2-QVx18-Com1	MVA-kQVx27	Empty MVA
**Animal GP4**								
~3 hr	NR	NR	NR	NR	NR	NR	NR	NR
8 hr	NR	eI 5mm	NR	NR	NR	NR	NR	eI 5mm
1	NR	eI 5mm	e 5mm	NR	e 5mm	NR	NR	eI 5mm
2	NR	EI 8mm	e 5mm	NR	NR	e dif	e 5mm	EI 8-10mm
3	NR	EI 10mm	eid	NR	NR	NR	NR	EI 8mm
4	NR	EI 10mm	eid	NR	NR	NR	NR	EI 8mm
5	NR	ei 10mm	ed	NR	NR	NR	NR	ei 5mm
6	NR	ei 8mm	ed	NR	NR	NR	NR	ei 5mm
7	NR	ei 5mm	ed	NR	NR	NR	NR	ei 5mm
**Animal GP5**								
~3 hr	NR	NR	NR	NR	NR	NR	NR	NR
8 hr	NR	NR	NR	NR	NR	e 5mm	NR	eI 5mm
1	NR	i	ed	NR	e 5mm	e 5mm	NR	eI 5mm
2	NR	ei 5mm	NR	NR	NR	e 3mm	e 3mm	EI 8-10mm
3	NR	EI diff	NR	NR	NR	e 3mm	NR	EI 7mm
4	NR	EI diff	NR	NR	NR	e 3mm	NR	EI 6mm
5	NR	ei	NR	NR	NR	e 3mm	NR	ei 6mm
6	NR	ei	NR	NR	NR	e 5mm	NR	ei 6mm

7	NR	ei	NR	NR	NR	e 4mm	NR	ei 6mm
**Animal GP6**								
~3 hr	NR	NR	NR	NR	NR	NR	NR	NR
8 hr	NR	NR	NR	NR	NR	NR	NR	eI 5mm
1	NR	e 5mm	eI 6mm	ed	e 6mm	e 6mm	e 5mm	eI 10mm
2	NR	e 6mm	e 6mm	NR	ed	e 3mm	e 3mm	eI 10mm
3	NR	EI 7mm	ed	NR	ed	NR	e	EI 8-9mm
4	NR	EI 6mm	ed	NR	ed	NR	e	EI 7mm
5	NR	EI 6mm	ed	NR	ed	NR	NR	EI 7mm
6	NR	EI 6mm	ed	NR	ed	ed	NR	EI 7mm
7	NR	EI 6mm	NR	NR	NR	NR	NR	ei 3mm

Table S3. MHC genotypes of immunogenicity study cynomolgus macaques. Samples from the pre-vaccination blood draws were sent to the Wisconsin National Primate Research Center (WNPRC) for MHC genotyping. Genotypes were determined by sequencing of genomic PCR amplicons of MHC class I and MHC class II DRB, DQA, DQB, DPA, & DPB sequences from the highly polymorphic peptide binding domain encoded by exon 2 of the MHC class I and class II loci. The sequence reads for each animal are aligned (mapped read count) against a custom database of Mauritian cynomolgus sequences, and the MHC haplotypes inferred based on the profile of alleles that are detected. Further background for interpretation of NHP MHC genotypes is available on the WNPRC web site: https://www.primate.wisc.edu/wp-content/uploads/2020/02/GS_WhitePaper-MHC-02202020.pdf
The single MHC haplotype shared amongst three of the four study animals is *italicized*.

**Prime vaccine**	**QVx27**	**QVx18-Com1**	**QVx27**	**QVx18-Com1**
**NHP ID**	**9119**	**9219**	**9319**	**9419**
**mapped_read_count**	25,751	6,624	37,523	9,729
**MHC-A Haplotype 1**	M6A	*M1A*	*M1A*	*M1A*
**MHC-A Haplotype 2**	M2A	*M1A*	M2A	M2A
**MHC-B Haplotype 1**	M6B	*M1B*	*M1B*	*M1B*
**MHC-B Haplotype 2**	M5B	*M1B*	M2B	M3B
**MHC-DRB Haplotype 1**	M6DR	*M1DR*	*M1DR*	*M1DR*
**MHC-DRB Haplotype 2**	M5DR	*M1DR*	M2DR	M3DR
**MHC-DQA Haplotype 1**	M6DQ	*M1DQ*	*M1DQ*	*M1DQ*
**MHC-DQA Haplotype 2**	M5DQ	*M1DQ*	M2DQ	M3DQ
**MHC-DQB Haplotype 1**	M6DQ	*M1DQ*	*M1DQ*	*M1DQ*
**MHC-DQB Haplotype 2**	M5DQ	*M1DQ*	M2DQ	M3DQ
**MHC-DPA Haplotype 1**	M6DP	*M1DP*	*M1DP*	*M1DP*
**MHC-DPA Haplotype 2**	M5DP	*M1DP*	M2DP	M3DP
**MHC-DPB Haplotype 1**	M6DP	*M1DP*	*M1DP*	*M1DP*
**MHC-DPB Haplotype 2**	M5DP	*M1DP*	M2DP	M3DP
